# Production and Downstream Integration of 5-(Chloromethyl)furfural from Lignocellulose

**DOI:** 10.1021/acssuschemeng.3c05525

**Published:** 2023-12-01

**Authors:** Jorge Bueno Moron, Gerard van Klink, Gert-Jan M. Gruter

**Affiliations:** † Van‘t Hoff Institute for Molecular Sciences, University of Amsterdam, Science Park 904, 1090 GD Amsterdam, The Netherlands; ‡ Avantium Chemicals BV, Zekeringstraat 29, 1014 BV Amsterdam, The Netherlands

**Keywords:** biorefinery, carbon utilization, downstream integration, lignocellulose, cellulose hydrolysis, CMF, furfural

## Abstract

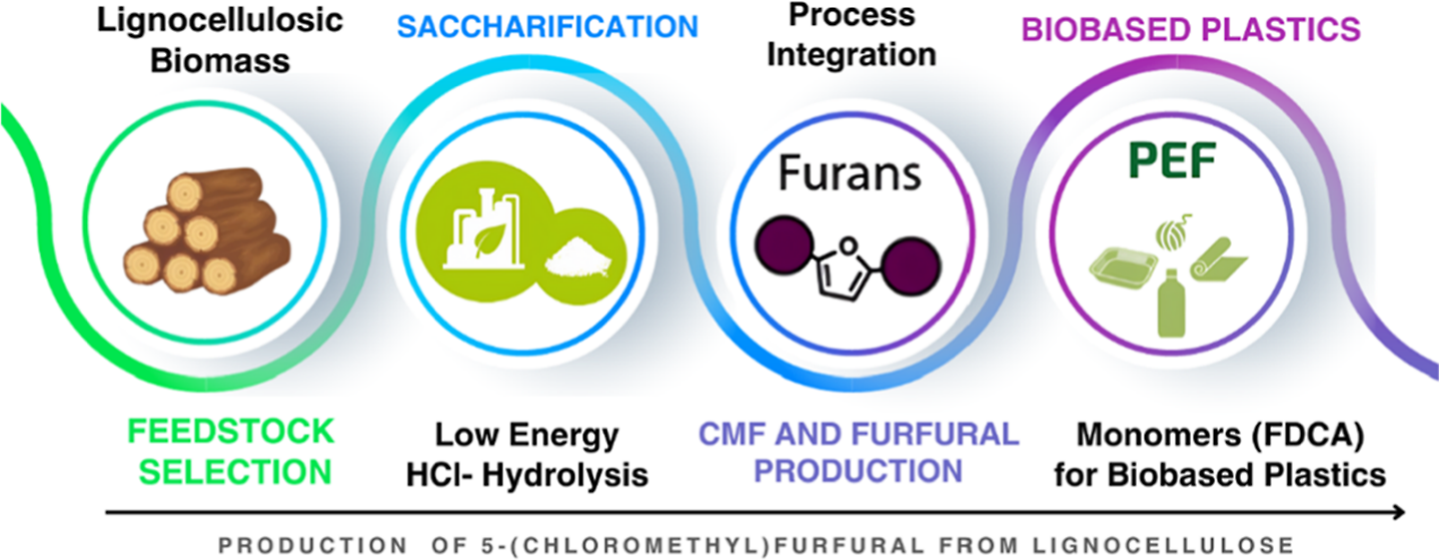

The importance of reducing the strong dependence of the chemical industry on fossil feedstock is no longer a debate. Above-the-ground carbon is abundant, but scalable technologies to supply alternatives to fossil-fuel-derived chemicals and/or materials at the world scale are still not available. Lignocellulosic biomass is the most available carbon source, and a first requirement for its valorization is the complete saccharification of its sugar-bearing components. HCl-based technologies can achieve this at 20 °C and ambient pressure. These principles were disclosed in the 1920s, but the inability to economically separate sugars from acids impeded its commercialization. Avantium Chemicals B.V. developed a modern version of this “Bergius” highly concentrated acid hydrolysis, in which the saccharides in HCl are transformed into furanics without any prior purification, in particular, to 5-(chloromethyl)furfural (CMF). Saccharide conversion to CMF was developed by Mascal in the early 2000s. CMF is extracted in situ using immiscible organic solvents, allowing for an easy product separation. This study not only targets to investigate the viability and optimization of this integrated process but also aims to predict the outcome of the CMF formation reaction by applying design of experiment techniques from the hydrolyzed saccharides varying a broad range of reaction parameters.

## Abstract 2

We have integrated our wood-based biorefinery with a subsequent conversion and separation to CMF as an intermediate for our PEF polyester.

## Introduction

In 2023, the Intergovernmental Panel on Climate Change (IPCC) showed in its latest report that the global surface temperature raised by 1.1 °C from 2011 to 2020 as compared to the period 1850–1900.^1,2^ Global greenhouse gas (GHG) emissions have continued to increase, with contributions from unsustainable energy and material consumption and production.

One of the reasons for global warming is the emission of carbon dioxide (CO_2_), which has significantly increased by consuming and burning fossil fuels to produce chemical products and fuels for modern society.^3^ It is expected that GHG emissions in 2030 will increase the global surface temperature by more than 1.5 °C, and it will be hard to limit this warming to 2 °C.^2^ Cumulative carbon emissions and the level of GHG emissions largely determine whether global warming can be limited to 1.5 or 2 °C. In this regard, the solution starts by developing chemical processes that solely depend on carbon sources from above the ground. Natural biomass- or plant-derived chemical products and fuels are considered carbon neutral when produced with renewable energy because plants absorb CO_2_ during their growth, and the total CO_2_ emission from the cradle to the grave is not adding new CO_2_ into the atmosphere.

**Figure 1:**
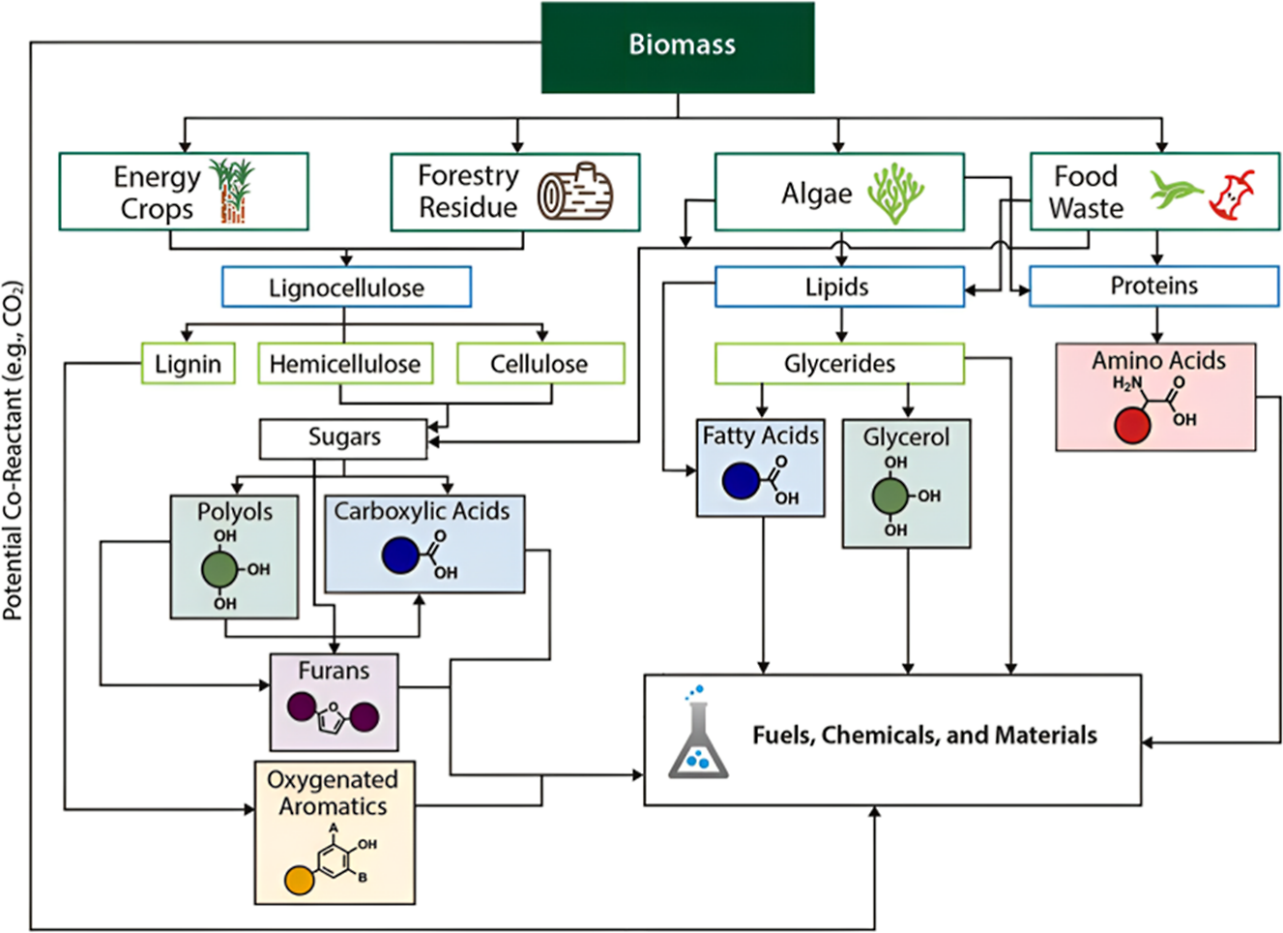
Biomass utilization and potential in replacing fossil feedstock resources for the production of fuels, chemicals, and biobased materials (reproduced from Mujtaba et al.^9^).

Therefore, developing a technology for the complete valorization of lignocellulosic biomass into chemical products will mitigate the worst scenarios of global warming.^3^ For more than a century, several companies tried to meet this need.^4−8^ Biorefineries fed with agricultural residues developed and transformed large volumes of lignocellulosic biomass in a wide portfolio of bioproducts (Figure 1).^9^ However, the commercialization of biorefineries fed on lignocellulosic biomass to provide world-scale alternatives to the petrochemical industry is still an economical challenge.

Governmental institutions and companies tried to commercialize the saccharification of lignocellulosic biomass but failed on achieving long-term production and stable plant operation.^10−16^ Within the most remarkable industrialization attempts, the process developed by the German chemist Friedrich Bergius in the 1920s stands out.^16,17^ His vision to supply the world with nonfood (second generation; 2G) sugars for the production of cattle food, chemicals, and biobased materials was set to work.^18^ This process used highly concentrated aqueous hydrochloric acid (HCl) solutions (40 wt %) at ambient temperatures to achieve full lignocellulose hydrolysis, while minimizing the utilization of energy.^8,11,19^ Despite this, the lack of modern equipment to withstand corrosion, the inconsistent feedstock supply, and the Second World War forced Bergius to close the factories.^20^

**Figure 2:**
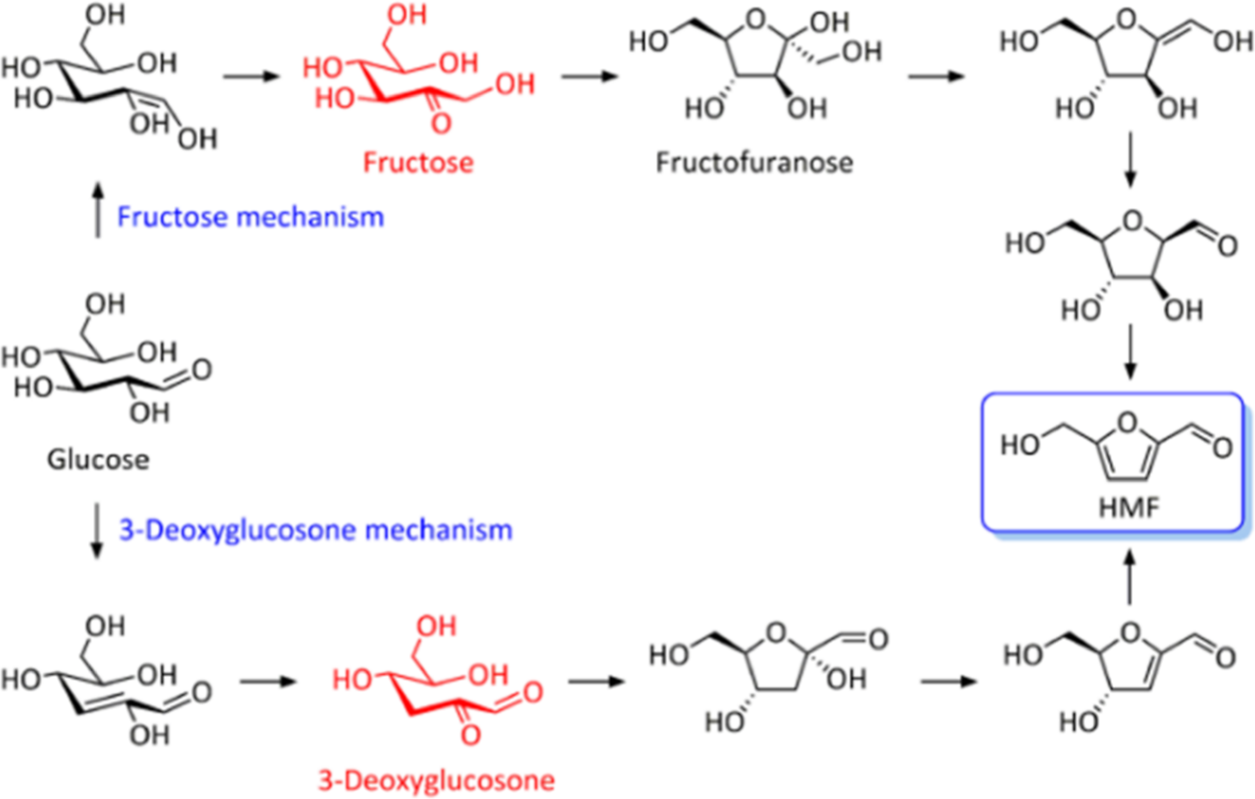
Possible pathways for the acidic conversion of glucose to HMF (from Zhu et al.^24^).

Avantium (The Netherlands), developed during the 2010–2020s, is a modern version of this saccharification.^21,22^ The downstream challenges were still present from the utilization of HCl solutions in which the hydrolyzed sugars can easily decompose during the acid–sugar separation. However, integrating a subsequent step for the conversion of the carbohydrates (in solution) into other molecules, which are easier to separate from the acids, simplified the downstream process. Furanic compounds derived from the dehydration of sugars have become excellent platform molecules for the production of biobased fuels and materials. Under the right acidic conditions and at a moderately elevated temperature, glucose quickly transformed to 5-(hydroxymethyl)furfural (HMF) via a dehydration reaction in HCl^23^ (Figure 2). HMF is a very valuable platform chemical in the chemical industry, but it lacks thermal and chemical stability.^23,24^

The halogenated analogues of HMF, commonly referred to as halomethyl furfural (XMF, X = F, Cl, Br, and I), provide longer shelf storage compared to their hydroxy analogue. The lower hydrophilicity resulting from the halogen in the XMF molecule provides higher thermal and chemical stability. HMF quickly interconverts to 5-(chloromethyl)furfural (CMF) in the presence of concentrated HCl. As CMF has a lower polarity than its hydroxy analogue HMF, it allows extraction of CMF from the aqueous acidic solution using immiscible organic solvents. The basis and principles of this CMF extraction and production from biomass and saccharide solutions were developed and extensively investigated by Mascal and Nikitin in the early 2000s.^25−28^

**Figure 3:**
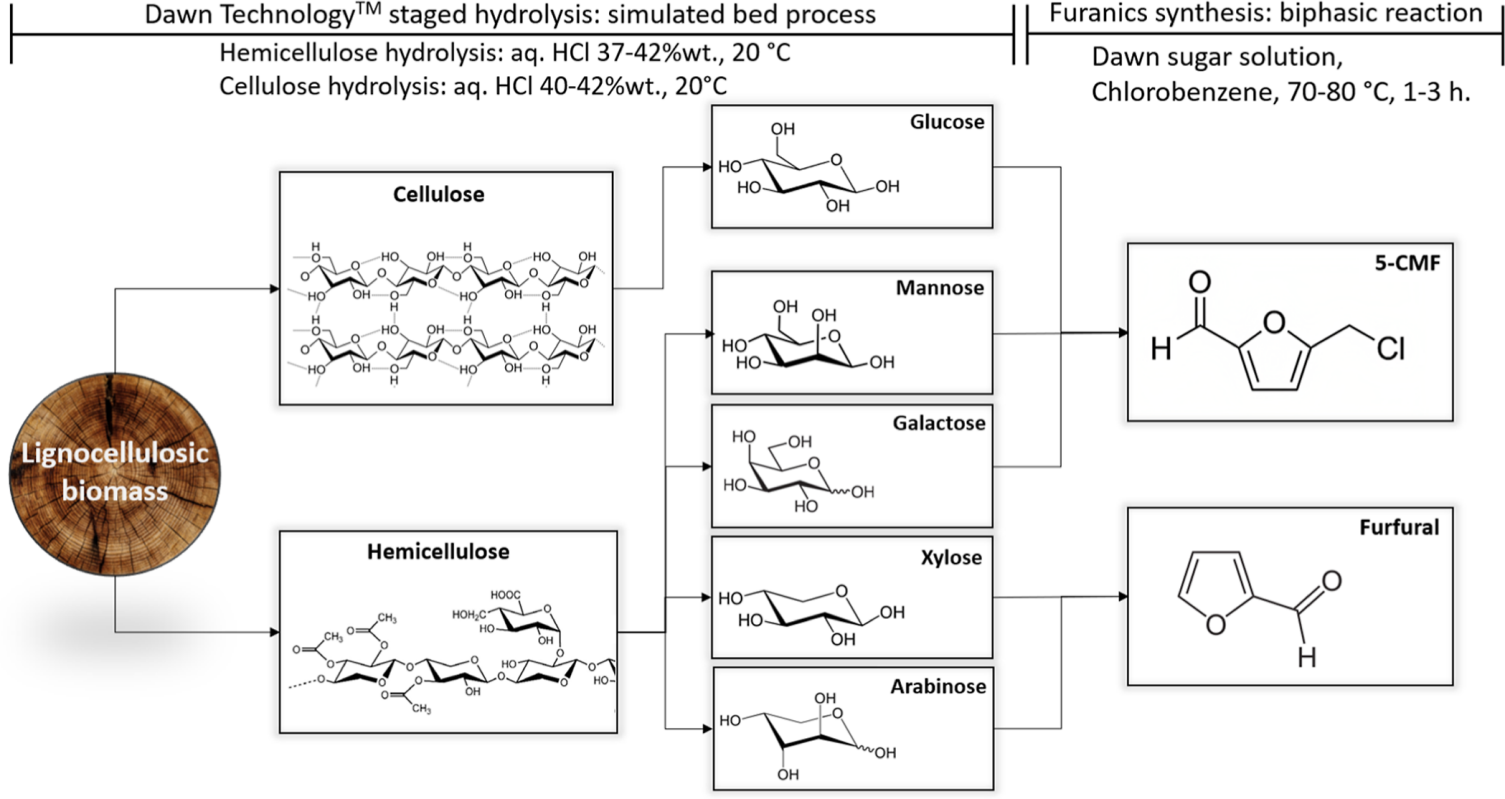
Lignocellulosic conversion to furanics in an integrated two-step process developed by Avantium.^29^

The CMF extraction into an immiscible organic solvent occurs in situ using biphasic reaction media, which contains the saccharide–HCl solution and the immiscible solvent. Mascal developed this technique, allowing him to isolate CMF in high yields from acidic cellulose solutions.^25^ The process integration is shown in Figure 3. This technology allows for easier separation of the final product from the solvent, solving many downstream problems of saccharification biorefineries. The aqueous HCl solution is recycled for the continuous saccharification of new lignocellulose, and the organic phase is evaporated from the furanics to start a new cycle of CMF production.^29^

These furanic compounds are versatile building blocks used for the production of biobased materials, shown, for instance, in the synthesis of furan-2,5-dicarboxylic acid in the obtention of polyethylene 2,5-furandicarboxylate, a renewable alternative to polyethylene terephthalate with improved physical properties for the food and beverage industry.^30,31^ An integrated process for the continuous valorization of lignocellulosic biomass into biobased furanics was developed first to reduce the dependence of the chemical industry to fossil fuels in the production of chemicals and materials and second to meet the goals sets for 2030 by the IPCC to reduce the carbon footprint and the CO_2_ emissions of the chemical industry.

## Materials and Methods

### Materials

#### Reagents and Solvents

Hydrochloric acid (HCl) solution in water (37 wt %), tridecane (>99%), chlorobenzene (99.6%), fluorobenzene (99%), bromobenzene (>99%), anisole (99%), 2-chloroanisole (98%), 1,2-dichloroethane (99.5%), *o*-difluorobenzene (>98%), toluene (99%), 2-phenyldodecane (97%), and dodecane (99%) were purchased from Fisher Scientific Solvents (technical grade). Lignocellulosic biomass (Aspen wood chips) was provided by Staatsbosbeheer, grown and collected in The Netherlands.

HCl solution in water (42 wt %) was produced by absorbing 100% HCl gas in a 32 wt % HCl solution. This was done at room temperature and 7 bar where the 32 wt % HCl solution entered from the top, and 100% HCl gas entered via an adsorption column (graphite column lined with PTFE) at the bottom of an absorber chamber. Midway through the adsorption column, the HCl concentration is recirculated to maximize stirring and provide a homogeneous HCl concentration throughout. Unabsorbed HCl gas re-enters the 36 wt % solution at the top of the adsorption column. At the end of the cycle, the 42 wt % HCl solution was cooled to 5 °C and reduced to atmospheric pressure. The molarity of HCl was determined in triplicate by a titrimetric analysis at 25 °C using an 809 Titrando unit (Metrohm AG). 1 mL of HCl was diluted in 100 mL of demineralized water, after which a solution of 1.0 M NaOH was used to determine the HCl concentration. The mass percentage of HCl was calculated based on the mass of the titrated sample.

### Equipment

#### Saccharification of Biomass

Reactors of 213 L constructed from fiber-reinforced plastic with a 3 mm polyvinyl chloride lining. Each reactor has a conical bottom with a granular sieve below for liquid product off-take and a Hastelloy full-bore (segmented) ball valve. The reactor system is designed as a simulated moving bed of seven identical reactors in two parallel trains. Each reactor is operated in switch mode, in which seven phases of 8 h can be distinguished.

#### CMF and Furfural Synthesis

The reactions were performed in 9 mL Ace glass high-pressure tubes purchased from Sigma-Aldrich (product code: 8648-17) and were used in aluminum heating blocks custom-made by Observator Precisietechniek B.V.

### Characterization Methods

#### Ion-Exchange Chromatography

Ion-exchange chromatography (IC) was used for the quantification of soluble monosaccharides using a Dionex ICS-5000 system with a CarboPac PA1 Analytical Anion Exchange Column (2 × 250 mm) and a pulsed amperometric detector. The mobile phase consisted of a mixture of Milli-Q water and 0.1 M sodium hydroxide (NaOH).

#### High-Performance Liquid Chromatography

High-performance liquid chromatography (HPLC) was used to determine the concentration of sugar degradation products. HPLC was performed on an Agilent 1260 Infinity II system with the 1260 series refractive index and diode array (DAD WR) detectors. The column was an Aminex HPX-87H (300 × 7.8 mm; dp 9 μm) using 5 mM sulfuric acid (H_2_SO_4_) in Milli-Q water for the mobile phase.

#### Gas Chromatography

The final product yield of CMF was identified using gas an Agilent 5975C gas chromatograph (GC) with a triple-axis mass selective detector. The system was equipped with an Agilent J&W DB624 column (20 m × 0.18 mm × 1 μm) using helium as the carrier gas. CMF and degradation products were quantified with a Thermo Scientific Trace 1310 GC equipped with a flame ionization detection (FID) Agilent J&W DB-624 UI (30 m × 0.25 mm × 1.4 μm) and helium for separation.

#### ^1^H NMR Spectroscopy

^1^H nuclear magnetic resonance spectroscopy (^1^H NMR) analysis was performed on a Bruker Avance III HD (600 MHz). Samples were dissolved in deuterated chloroform (CDCl_3_).

### Pilot Plant Synthesis of Prehydrolysate (Hemicellulose Saccharification)

In a typical procedure, eight reactors filled with 50 kg of dried Aspen wood chips (moisture content <10 wt %) were connected in series. The first reactor was filled for 1 h with a 37 wt % aqueous HCl solution in a ratio of 3 g/g (37 wt % HCl/dried Aspen wood chips) at 20 °C. Then, additional 37 wt % HCl was pumped into the first reactor to fill the other seven reactors connected in series using the same 3 g/g ratio (37 wt % HCl/dried Aspen wood chips). During this procedure, the HCl solution with hemicellulosic sugars moves across the eight reactors connected in series, and the product stream referred to as prehydrolysate is collected from the last reactor. After every reactor was in contact with 37 wt % HCl for 24 h, tridecane was fed to push the remaining prehydrolysate out of the reactors. Reference materials and samples were analyzed on HPLC and IC to identify and quantify the compounds in the sugar hydrolysate and degradation composition.

### Pilot Plant Synthesis of Main Hydrolysate (Cellulose Saccharification)

In a typical procedure, eight reactors at 20 °C filled with residual lignocellulose and tridecane after the prehydrolysis were connected in series. The first reactor in series was continuously pumped with an aqueous 42 wt % HCl solution in a ratio of 2 g/g (42 wt % HCl/starting dried Aspen wood chips) at 5 °C. The tridecane inside the reactors was pushed to the reactors connected in series, followed by the 42 wt % HCl-containing cellulosic sugars in it. During this time, the HCl solution with cellulosic sugars in it raised to a 20 °C controlled reactor temperature, and this effluent moved across the eight reactors connected in series, with the product stream referred to as the main hydrolysate being collected from the last reactor. After every reactor was in contact with 42 wt % HCl for 24 h, tridecane was fed to every reactor to push the remaining main hydrolysate outside of it. Reference materials and samples were analyzed on HPLC and IC to identify and quantify the compounds in the sugar hydrolysate and degradation composition.

### Synthesis of Furfural from Prehydrolysate

In a typical procedure, 1 mL of prehydrolysate and 3 mL of the organic solvent of choice (chlorobenzene, fluorobenzene, bromobenzene, anisole, 2-chloroanisole, 1,2-dichloroethane, *o*-difluorobenzene, toluene, 2-phenyldodecane, or dodecane) were introduced in a single Ace glass high-pressure rated tube. The reaction was carried out at 80 °C for 1.5 h under magnetic stirring. After completion, the reactor was cooled to room temperature. The organic phase was then collected, and the remaining aqueous phase was washed with an additional 3 mL of the organic solvent of choice for 1 h under magnetic stirring at room temperature. The organic layer was isolated, and the washing step was repeated twice. The organic layers from the reaction and the two washing steps were combined. NMR and GC–MS analyses were performed and compared to NIST 11 libraries to confirm compound identities. GC-FID with reference materials and internal standards was used to quantify their concentration.

### Synthesis of CMF from Main Hydrolysate

In a typical procedure, 1 mL of main hydrolysate and 3 mL of the organic solvent of choice (chlorobenzene, fluorobenzene, bromobenzene, anisole, 2-chloroanisole, 1,2-dichloroethane, *o*-difluorobenzene, toluene, 2-phenyldodecane, or dodecane) were introduced in a single Ace glass high-pressure rated tube. The reaction was carried out at 80 °C for 3 h under magnetic stirring. After completion, the reactor was cooled to room temperature. The organic phase was then collected, and the remaining aqueous phase was washed with an additional 3 mL of the organic solvent of choice for 1 h under magnetic stirring at room temperature. The organic layer was isolated, and the washing step was repeated twice. The organic layers from the reaction and the two washing steps were combined. NMR and GC–MS analyses were performed and compared to NIST 11 libraries to confirm compound identities. GC-FID with reference materials and internal standards were used to quantify their concentration.

## Results and Discussion

### Feedstock Selection and Composition

From a macromolecular perspective, lignocellulosic materials contain three biopolymers: hemicellulose, cellulose, and lignin. The proportion of each component and the composition of the hemicellulose and lignin vary depending on the type of biomass (e.g., hardwood, softwood; corn stover, and bagasse) and growth conditions. Lignin is usually present binding with the cellulose and hemicellulose, and it differs from the other two (polysaccharide) biopolymers by its highly branched C6 aromatics containing an amorphous structure.^32^ Overall, softwoods and hardwoods have different lignin contents; with softwoods containing up to 34% and hardwood which has around 23–30% of lignin. The remaining macromolecular composition of the lignocellulose is commonly referred in the literature as holocellulose, which is composed by all the carbohydrates present in it.^33^ This is mainly the hemicellulose and the cellulose and usually corresponds to about 65–75 wt % of the total lignocellulose dry weight.^34^

The lower molecular weight substances in wood are extractives and ash. Wood extractives are the nonstructural components in wood, and depending on the wood type, they can constitute about 4–13% w/w of the wood.^35^ Extractives in wood are defined as a family of organic compounds that can easily be extracted from wood using neutral solvents.^36^ They are commonly found in the heartwood inside the trunk, and they protect the tree from environmental stress.^35^ According to their chemical composition, extractives can be divided into three major subgroups: aromatic phenolic compounds, aliphatic compounds (fats and waxes), and terpenoids.^37^

The solid residue after the thermal decomposition of wood is the ash. It usually constitutes less than 1% of the starting weight of the wood. A high percentage of Ca, K, P, Al, Si, and Fe can be found in it. High Fe content in the ash may result from trees located in highly urbanized areas. Sometimes, other minor but potentially toxic elements, like Pb, Cd, Zn, Ni, Cu, As, Hg, and Cr, are found in the ashes.^38^

**Figure 4:**
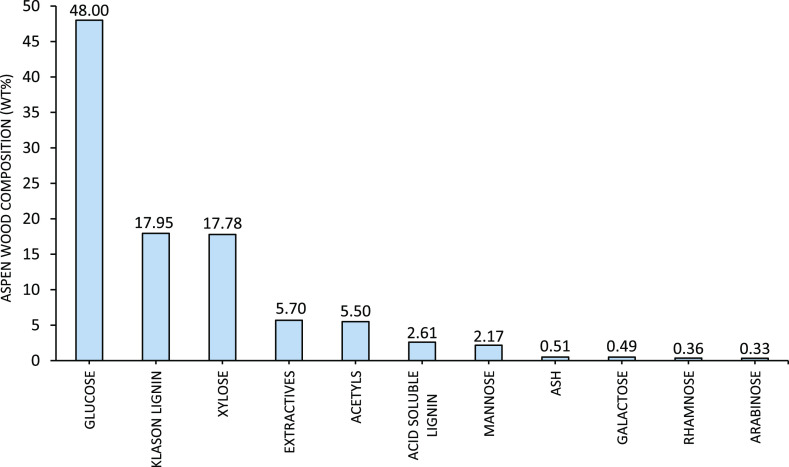
Chemical composition of Aspen wood chips, analysis by Celignis Biomass Analysis Laboratory.

The feedstock used during this study was Aspen wood chips, grown and collected in The Netherlands by Staatsbosbeheer, the Dutch forestry commission. The chemical composition of this wood material was analyzed by Celignis Biomass Analysis Laboratory, located in Ireland, and the data are shown in Figure 4.

### DAWN Saccharification Technology

DAWN technology is Avantium’s modern modification of the original Bergius saccharification process, whose principles and details were first patented in 1917.^8,39^ The Bergius–Rheinau, or Bergius–Willstätter–Zechmeister process, is the hydrolysis of biomass by the action of fuming hydrochloric acid at a low temperature in one single step. This technology was developed to alleviate the scarcity of food during the First World War by transforming wood waste produced by pulp and wood distillation industries into edible sugars. For that, Bergius adapted the saccharification of wood to obtain a concentrated and digestible sugar, or a mixture of sugars, fit for human consumption or to be used as cattle food.^40^

**Figure 5:**
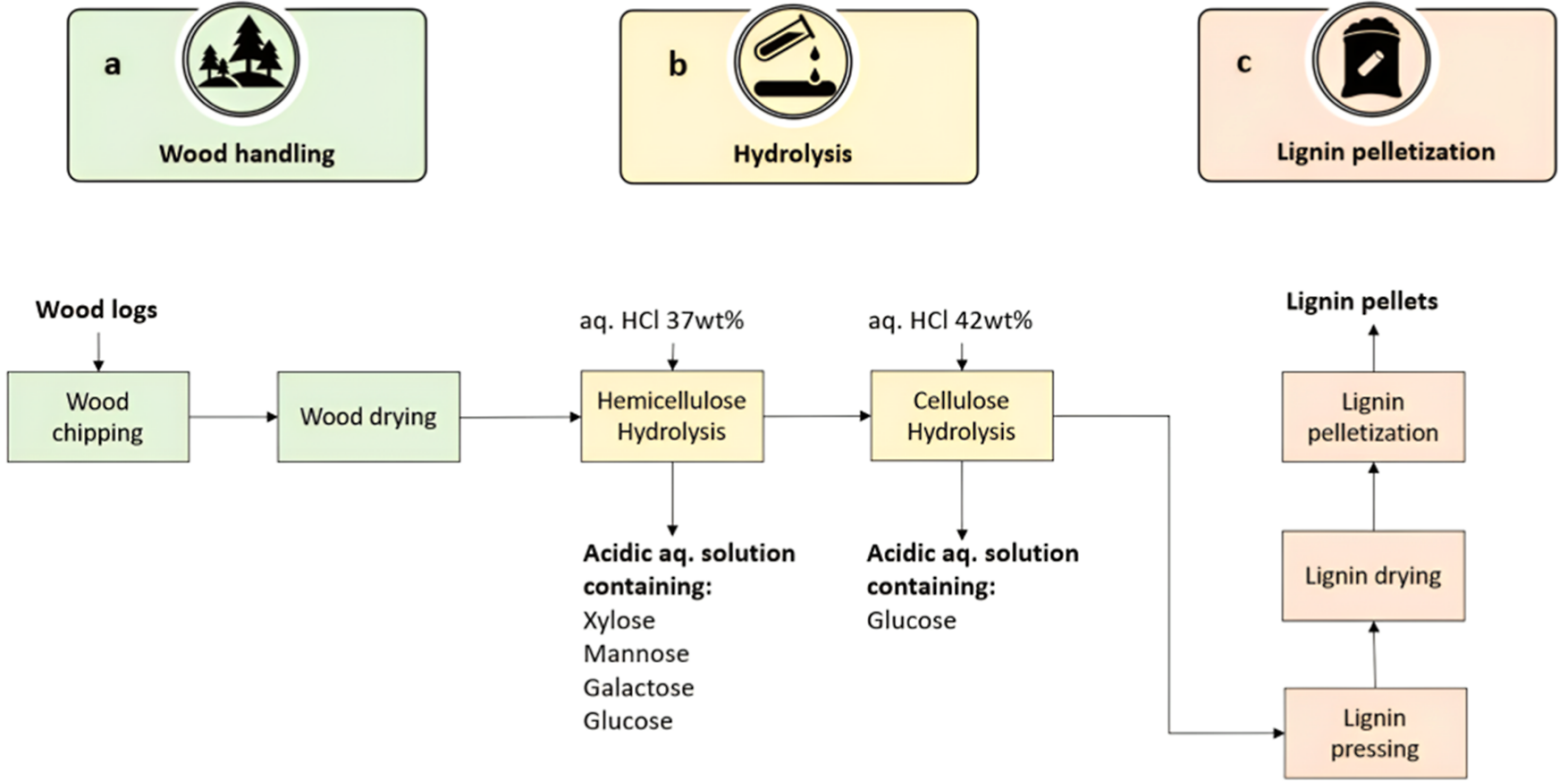
Schematic representation of the DAWN technology. The process is divided into three stages, namely, (a) wood handling (debarking, chipping, and drying; green), (b) selective hydrolysis (yellow), and (c) lignin pressing, drying, and pelletization (red).

The Bergius saccharification process lacked the selectivity to hydrolyze and separate the different polysaccharides composing the biomass.^12,39^ Additionally, the lack of modern equipment to fully recover the acid made the overall economic aspect of the process inefficient.^20^ Consequently, during the 1930–1970s, companies, governments, and institutions working with this technology had to cease operation within 20 years.^20^ After the oil crisis, the industry shifted toward enzymatic hydrolysis and milder saccharification technologies for the conversion of biomass.^41−43^ Later in the early 2010s, Avantium, located in The Netherlands, revisited the original Bergius saccharification process. Several modifications to this technology were patented^21,22,44−46^ by Avantium to achieve a selective and fractional saccharification of lignocellulosic biomass (Figure 5). This selective hydrolysis of biomass is termed the DAWN technology. It is a two-stage HCl hydrolysis, in which first the hemicellulose is recovered by placing the biomass in contact with aqueous 37 wt % HCl. This stage is referred to as prehydrolysis, and it is followed by the hydrolysis of cellulose with 42 wt % HCl, referred to as main hydrolysis. The acid–sugar separation and purification, common pitfalls in the past for the saccharification industry, can be avoided in the special DAWN process variation “YUKON” in which the acidic saccharide hydrolysate, obtained from the hydrolysis of biomass, is directly converted thermally into furanics furfural and CMF. This process integration and conversion to furanics are later disclosed and detailed in the furfural and CMF production section (vide infra).

**Figure 6:**
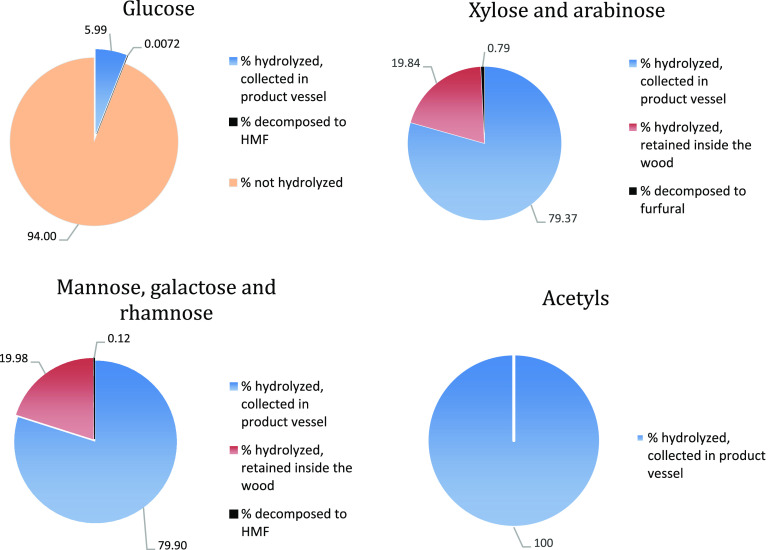
Prehydrolysis mass balance (wt %). Sugars hydrolyzed and collected in the product vessel are shown in blue. Sugars hydrolyzed and retained inside the biomass after the prehydrolysis are shown in orange. Sugars decomposed to HMF or furfural are shown in black. The fractions of the biomass that were not hydrolyzed during this stage are shown in green.

In this study, we focus on the YUKON process, which starts with the hydrolysate product produced in stage (b). The first and selective saccharification of the hemicellulose is achieved with 35–40 wt % HCl (depending on the residual moisture content of the lignocellulosic biomass feedstock; typically 5–10 wt %), and the porosity of the wood material increases. Even with quantitative yields in terms of saccharification, around 20% of the hemicellulosic sugars (Table 1) remains inside the solid particle at the end of the prehydrolysis. These sugars are later recovered during the first fractions of the main hydrolysis, producing a mixed sugar solution that is collected separately from the main hydrolysate. During the prehydrolysis, around 6% of the glucose present in the biomass is also hydrolyzed. Glucose may also be present in the hemicellulose, but small amounts of cellulosic glucose can also be brought into solution during this phase. Acetic acid, resulting from the scission of acetyl ester groups at the side chains of the hemicellulose, is also found in the prehydrolysate. Furfural and HMF from the decomposition of sugars are observed in solution, but in small quantities only (Table 1). The acidic product sugar hydrolysates are usually 1–2% less acidic than the starting acid concentration (37 wt % becomes 35 wt % in the hydrolysate). The remaining moisture after drying the wood is located in the deepest layers of wood tissue. This water does not dilute the acid immediately but rather slowly, and since the acid is continuously fed to the reactor, the overall acid concentration remains constant during the hydrolysis. The performance of the prehydrolysis in terms of sugar mass balance, yield of hydrolysis, and sugar decomposition compounds is shown in Figures 6 and 8.

**Table 1:** Analysis of the Water- and Ethanol-Soluble Extractives Found during the Saccharification of Aspen Wood Chips

	water-soluble extractives [wt %]	ethanol-soluble extractives [wt %]
prehydrolysate	1	0
main hydrolysate	0.05	0
lignin	25	100
decomposed	74	0

**Figure 7:**
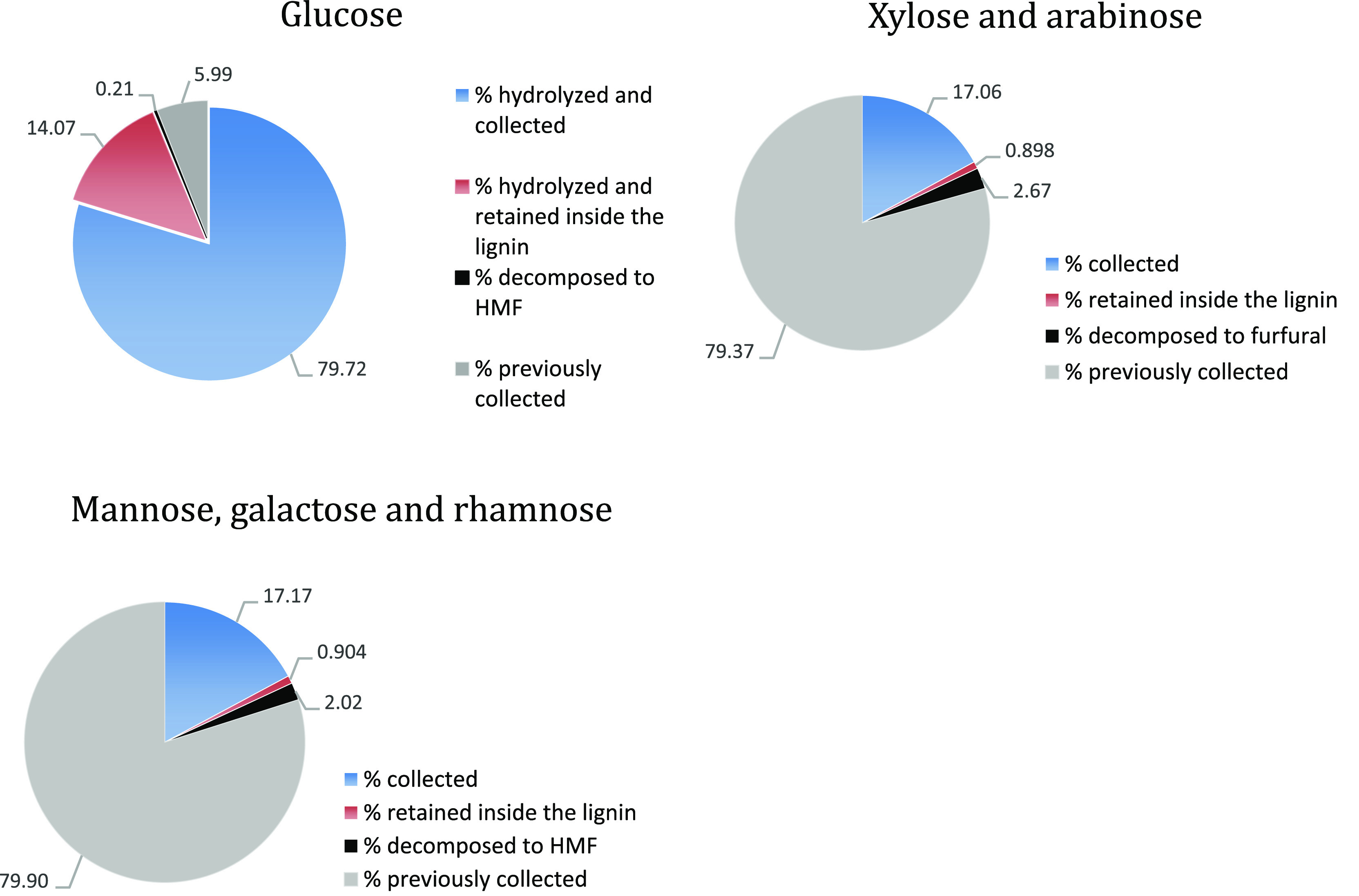
Main hydrolysis mass balance (wt %). Sugars hydrolyzed and collected in the product vessel are shown in blue. Sugars hydrolyzed but retained inside the lignin after the main hydrolysis are shown in orange. Sugars decomposed to HMF or furfural are shown in black. The sugars that were previously recovered during the prehydrolysis are shown in gray.

**Figure 8:**
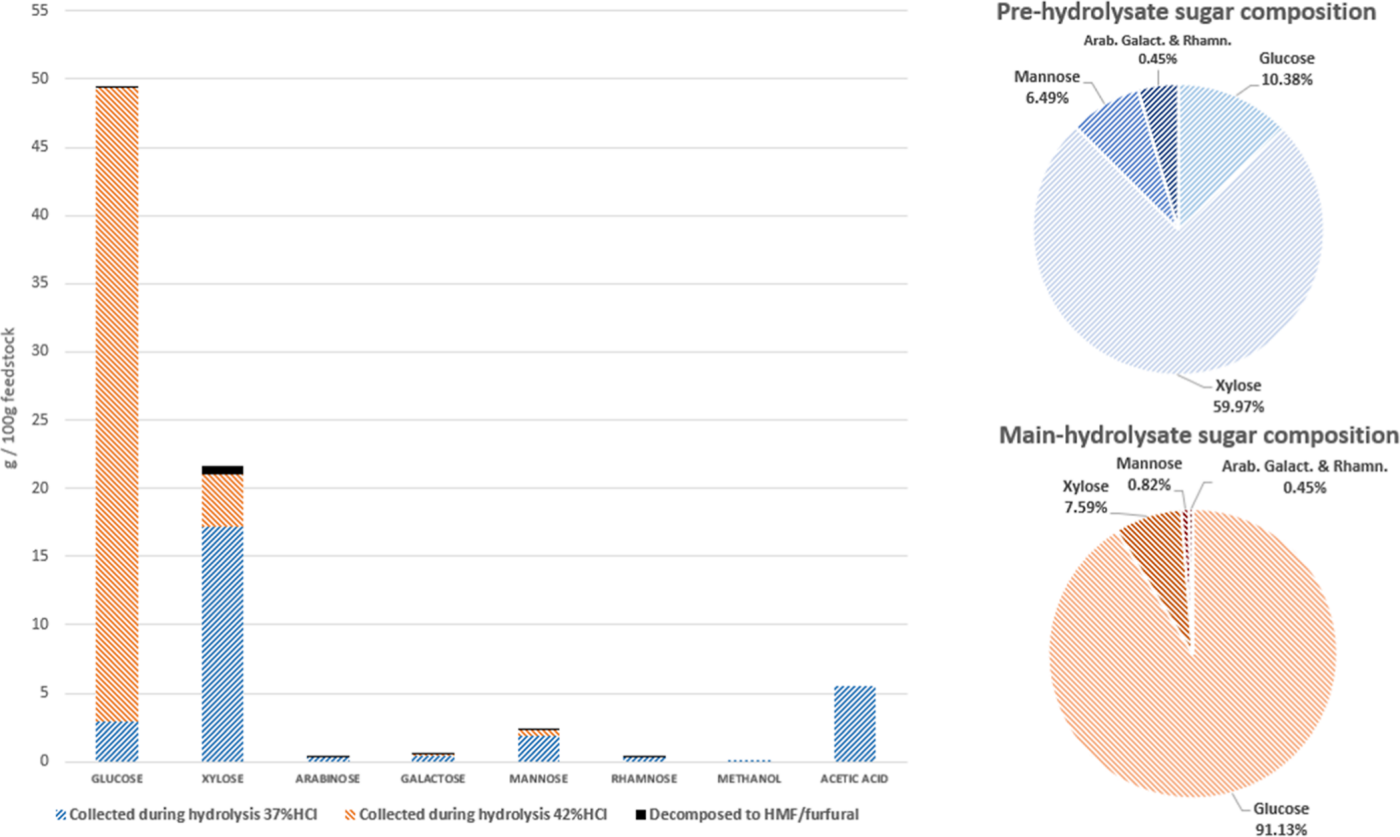
Left: sugar recovery during the hemicellulose prehydrolysis (37 wt % HCl) and the cellulose main hydrolysis (42 wt % HCl). Right: sugar composition of the product sugar solutions from the pre- and main hydrolysis.

The main hydrolysis is the second-stage saccharification of the more recalcitrant cellulose using concentrated aqueous 42 wt % HCl. The yield of hydrolysis in this stage is quantitative, and the removal of the cellulose from the wood structure further increases the porosity of the material. This leads to partial retention of the main hydrolysate solution inside the residual lignin. Also, a small fraction of hemicellulosic sugars retained from the prehydrolysis (approximately 2%) is decomposed to furfural and HMF during this stage. The remaining small fraction of retained sugars in the lignin is later recovered during the pressing and washing of the lignin. The performance of the main hydrolysis in terms of sugar mass balance, yield of hydrolysis, and sugar decomposition rates are shown in Figures 7 and 8.

During this study, some of the wood extractives were found in residual lignin and in different sugar solutions. The partitioning of the extractives in the hydrolysis is indicated in Table 1.

### Process Integration

The DAWN technology main challenge remains the efficiency to separate the sugars from the acidic solution. The main challenge is the high HCl concentration, in which the sugars can easily decompose above ambient temperature during their separation and purification. Furthermore, HCl and water form a negative azeotrope at 20.2 wt % HCl and 79.8 wt % water composition. This requires advanced separation techniques,^47,48^ such as dual-pressure distillation, which is expensive and complex to operate. Instead, in this study, the acid–sugar solutions from saccharification were heated directly to convert the sugars into furanics without any prior purification. The xylose present in the prehydrolysate can be converted to furfural, while the glucose from the main hydrolysate can be chemically transformed via acid-catalyzed dehydration to CMF. These furanic compounds are obtained using a biphasic system composed of the saccharide acidic solution and a nonmiscible organic solvent, in which the CMF and furfural were continuously extracted, limiting the contact time of these furanics with the strong acid present in the aqueous phase. By doing so, the separation of furanics in the organic solvent from the acid becomes a simple phase separation. Furfural and CMF can be obtained in high purity, while the organic solvent can be evaporated and recirculated back to the reactor.

The reaction conditions for the conversion of glucose to CMF and xylose to furfural are similar but not identical. Xylose requires lower temperatures and shorter reaction times to convert to furfural, compared to the conversion of glucose to CMF. This is of relevance when studying the sugar composition of the saccharide product streams of the DAWN process. The prehydrolysis (hemicellulose saccharification) mostly contains xylose, but small amounts of glucose are also present. This is similar to the sugar composition in the main hydrolysate, in which mostly glucose is present, but small fractions of hemicellulosic sugars can also be found. For this study, the simultaneous production of furfural and CMF was also evaluated, but ultimately, the furfural and CMF production processes were optimized separately. The prehydrolysate, mostly containing xylose, was used for the production of furfural, and CMF formed was considered a byproduct. The same philosophy was applied to the CMF reactor, fed with main hydrolysate from the hydrolysis of cellulose, and optimized to maximize the production of CMF from the glucose in it.

### Biphasic Systems

The conversion of saccharides using biphasic systems is a well-established method to produce CMF and furfural in high yields.^26,27,49−51^ The continuous extraction and protection of these furanics in an immiscible organic solvent drastically reduce the amount of side reactions between the furanics and the acid present in the aqueous phase.^25^ Haworth first reported in 1944 the use of biphasic systems to obtain CMF in 21.3% molar yield by mixing carbon tetrachloride with a saturated HCl aqueous solution of fructose.^52^ In 1978, Hamada obtained a patent reporting 77.5% CMF yield from hexose monosaccharides and disaccharides dissolved in an aqueous HCl solution in contact with toluene or carbon tetrachloride.^53^ In 1981, Szmant used similar reaction conditions achieving 92–95% CMF molar yield^54^ for the conversion of crystalline fructose present in an aqueous HCl solution in contact with chlorobenzene. Later, during the 2000s, Mascal and Nikitin set the basis^25−28,49^ to understand the reaction mechanism and parameters which impact the conversion of saccharides to CMF in biphasic systems. At an early stage of research, Mascal studied the conversion of monomeric sugars and microcrystalline cellulose but quickly expanded this research to biomass^27^ (corn stover or wood) and high oil content biomass, like soybean and sunflower seeds,^28^ achieving new milestones for obtaining CMF from (poly)saccharides.

Origin Materials (formerly MicroMidas; Sacramento, USA) announced in June 2023 the startup of the world’s first commercial CMF plant, located in Sania, Ontario. Their process allows for the production of CMF from lignocellulosic biomass in two separate steps.^55−57^ First, lignocellulosic biomass is mixed with HCl gas in a fluidized bed reactor for a quick first hydrolysis. The subsequent second step comprises a washing with a Lewis acid (e.g., LiCl and CaCl_2_) solution in dichloromethane (DCM) (or a similar organic solvent) to produce CMF in the DCM solution. This differs from the process developed later by Avantium,^29^ in which the milder conditions for hydrolysis, using room temperature and ambient pressure, allow for a first selective saccharification of the biomass components and separation from the lignin. Later, the excess of HCl present in the acidic sugar solution after hydrolysis is enough to produce CMF in high yields using halogenated organic solvents (e.g., chlorobenzene) in biphasic reactors.

### Mechanism of Formation

**Figure 9:**
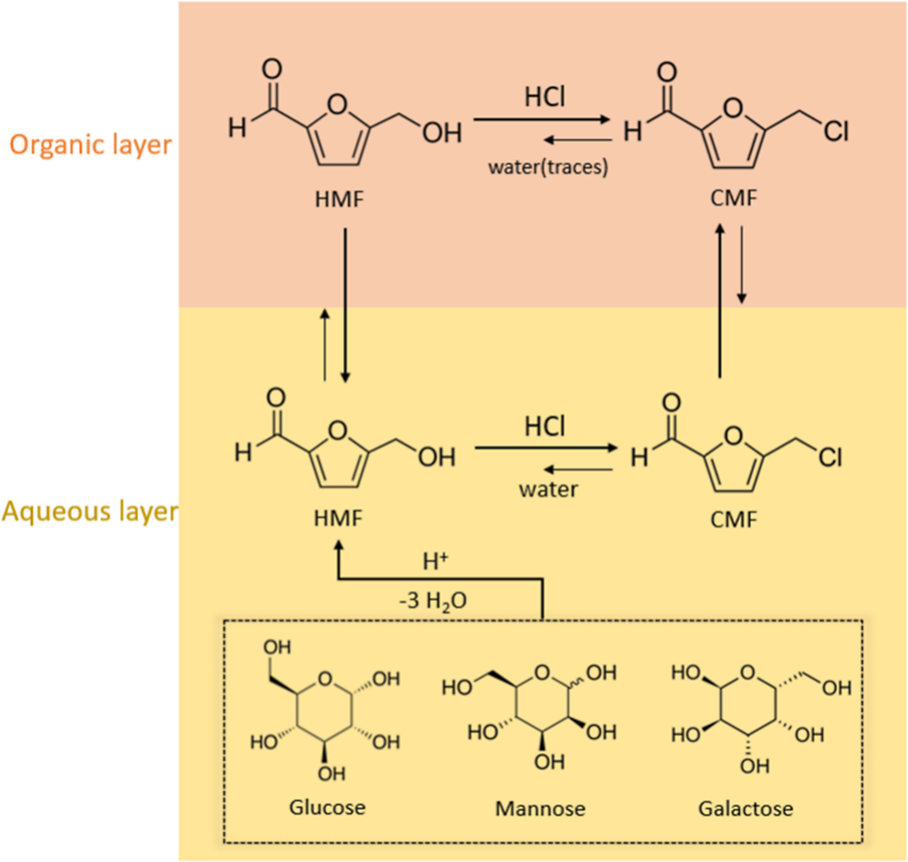
Conversion of C_6_ lignocellulosic sugars to CMF using biphasic systems. Yellow represents the aqueous phase containing the C_6_ sugars in a concentrated aqueous HCl solution. Orange represents the organic phase (assuming lower density), where the HMF and CMF are continuously extracted and protected from the acid solution. Overall, CMF yields benefit from biphasic systems in which the organic layer can efficiently extract both CMF and HMF. Ring-opening and polycondensation reactions from the decomposition of sugars, HMF, and CMF result in the formation of humins,^60^ which mostly stay insoluble in the water layer, but they can also be found in the organic phase in smaller quantities.

The formation of CMF from C_6_ sugars in HCl is divided into two steps (Figure 9), namely, (a) C_6_ sugar dehydration to HMF and (b) halogenation of HMF to produce CMF. The acyclic conversion of glucose to HMF through fructose isomerization is the most commonly suggested pathway,^58^ but other cyclic and acyclic routes in which glucose directly dehydrates into HMF are also reported.^58^ Another study^59^ showed that CMF yields benefit from organic solvents with affinity to extract HMF from the aqueous phase. HMF and CMF coexist in equilibrium, and they can both interexchange in the presence of water and HCl. It is suggested that a quick removal of the HMF from the aqueous HCl solution protects it from side reactions with HCl that lead to the formation of levulinic acid, formic acid, and polycondensation compounds like humins.^59,60^

**Figure 10:**
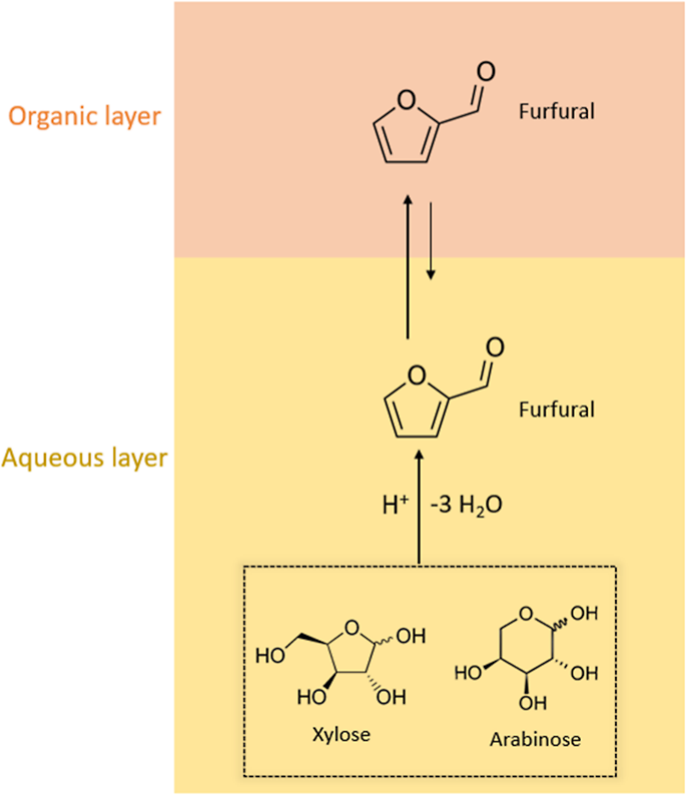
Conversion of C_5_ lignocellulosic sugars to furfural was performed using biphasic systems. Yellow represents the aqueous phase containing C_5_ sugars in concentrated aqueous HCl. Orange represents the organic phase (when having a lower density on top), in which furfural is continuously extracted and protected from the acid in the aqueous phase. Ring-opening and polycondensation side reactions, from the decomposition of the sugars and furfural, lead to humins, which are mostly found in the aqueous layer. Similar to CMF production, these side products can also be found in the organic layer but in smaller quantities.

The conversion of C_5_ hemicellulosic sugars xylose and arabinose into furfural is illustrated in Figure 10. The dehydration reaction occurs mostly in the aqueous phase in which this acid-catalyzed reaction produces furfural. It is advised to use organic solvents with slightly higher polarity compared to the conversion of hexoses to CMF since the hydrophilicity of furfural is higher and it could limit its extraction into a less-polar organic phase.^51^

### Reaction Parameter Analysis

Mascal et al. set the basis^26,28,59,61^ for understanding the impact of the reaction parameters that can be varied in the conversion of saccharides to CMF. Parameters with the greatest impact in the course of this reaction are categorized as follows: Hansen solvent parameters, solvent fraction, reaction temperature, acid concentration, reaction time, mass-transfer effect, and initial xylose and/or glucose concentration. These were primarily investigated during the period 2010–2020, either individually or in studies that combined two or more of these parameters. These studies were all performed using biphasic systems, which contained an acidic solution with the saccharide of the study or cellulosic material, together with an immiscible organic solvent. For our study, the saccharide solutions were the pilot plant product streams from the hydrolysis of Aspen woodchips with drying to 10% residual moisture as the only prior treatment. After the hydrolysis of hemicellulose and/or cellulose, the hydrolysate product stream (aqueous acidic saccharide solution) was placed in a sealed reactor with an organic immiscible solvent to form a biphasic system. This allows for the direct and continuous production of furfural and CMF depending on the hydrolysate composition. The DAWN pre- and main hydrolysates differ on their sugar composition and HCl concentration (vide supra).

### Solvent Selection

The role of the organic solvent is to selectively extract and protect the CMF and furfural formed during the dehydration of saccharides.^59,62^ The Hansen parameters allow one to study the miscibility between solvents^63^ and is a tool to predict the ability of these solvents to extract CMF and furfural in biphasic systems. The Hansen parameters of a solvent are given by the London dispersion forces, dipolar intermolecular forces, and hydrogen bond capacity. Lane et al.^59^ observed that over the course of reaction, the dispersion forces of the organic solvent had no impact in the CMF yield, while some hydrogen bond capacity was still necessary. The polarity of the organic solvent had the largest impact on its ability to extract HMF and CMF from the aqueous phase, protecting these two compounds from the highly concentrated acid solution. The correlation between the CMF yield and the solvent polarity was found to be *R*^2^ = 0.98, while the same correlation between the London dispersion forces and the CMF yield was lower but still relevant (*R*^2^ = 0.80).^59^

**Figure 11:**
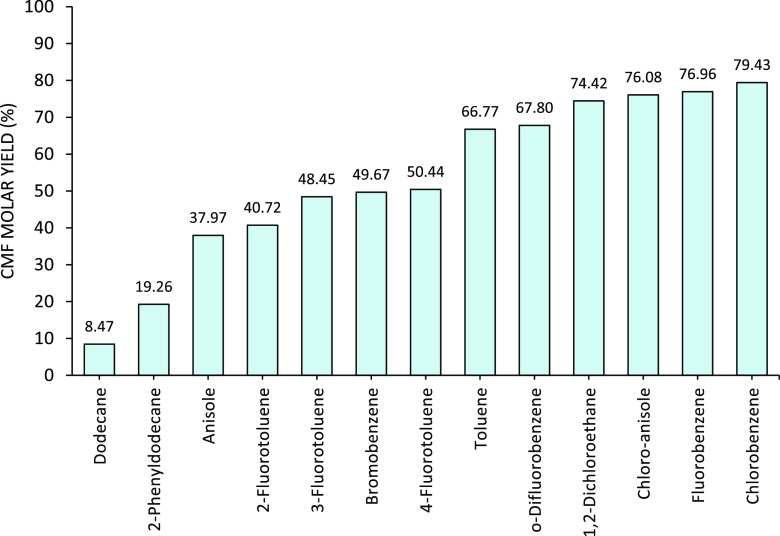
Solvent screening for the conversion of DAWN main hydrolysate (glucose oligomers) to CMF in biphasic systems. After 3 h at 80 °C, the reactors were cooled to room temperature. The organic layer was analyzed, and the results are shown in terms of CMF molar yield.

Based on the effect of the Hansen parameters on the CMF yield, a list of organic solvents (Table 2) was screened for the conversion of DAWN main hydrolysate (glucose oligomers) to CMF. The list of solvents covered a broad range of polarities while maintaining some hydrogen bonding capacity and London dispersion forces. The reactors were loaded with main hydrolysate (aqueous phase) and the organic solvent of choice from Table 2 in a 1:3 v/v ratio of hydrolysate/organic phase. These screening reactions were carried out in batch at 80 °C and for 3 h based on previous recommendations from Mascal et al.^26,28,59,61^ After completion, the reactors were brought to room temperature, and the organic phase was separated for analysis. The CMF yield obtained for each of the solvents screened is shown in Figure 11.

**Table 2:** Hansen Parameters of the Solvents Screened during the Conversion of DAWN Main Hydrolysate (Glucose Oligomers) to CMF^64,65^

entry	organic solvent	CMF yield [%]	London dispersion forces (δ_D_) [MPa^1/2^]	permanent dipole force (δ_P_) [MPa^1/2^]	hydrogen bonding (δ_H_) [MPa^1/2^]	source
1	chlorobenzene	79.4	19.0	4.3	2.0	(64)
2	fluorobenzene	77.0	18.7	6.1	2.0	(64)
3	2-chloroanisole	76.1	19.6	7.8	6.7	(65)
4	1,2-dichloroethane	74.4	19.0	7.4	4.1	(64)
5	*o*-difluorobenzene	67.8	18.0	9.0	1.0	(65)
6	toluene	66.8	18.0	1.4	2.0	(64)
7	bromobenzene	49.7	20.5	5.5	4.1	(64)
8	anisole	38.0	17.8	4.4	6.9	(65)
9	2-phenyldodecane	19.3				
10	dodecane	8.5	16.0	0.0	0.0	(65)

Chlorobenzene, fluorobenzene, 2-chloroanisole, and 1,2-dichloroethane showed higher CMF yields (Table 2, entries 1–4). The correlation between the Hansen parameters and the CMF yield is predictable, and the outcome of these experiments is aligned with previous conclusions of Lane and Mascal.^59^ It was observed that a high permanent dipole moment and good hydrogen bonding capacity are required to obtain good CMF yields, while the London dispersion forces were less relevant. This is also found in the reaction using dodecane (Table 2, entry 10), which has a permanent dipole of 0 MPa^1/2^, and it achieved the lowest CMF yield (8.5%). However, organic solvents with extremely high dipolar moment (Table 2, entry 5) can also extract other polar compounds from the aqueous phase, which can lead to undesired reactions with CMF and furfural.

**Figure 12:**
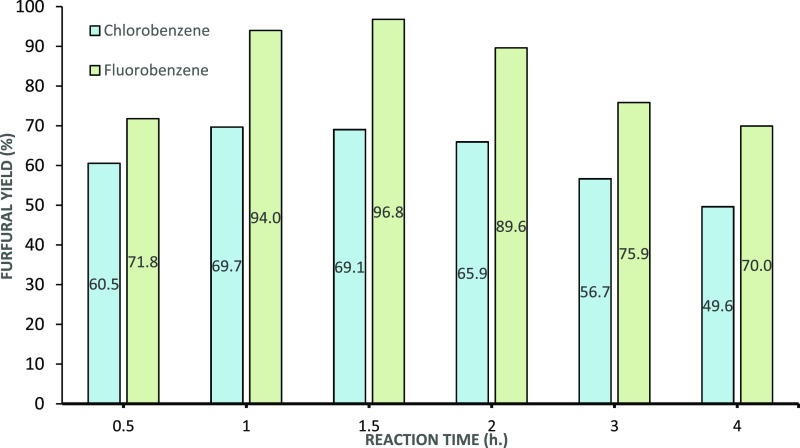
DAWN prehydrolysis (xylose oligomers) conversion to furfural at 80 °C in batch, with aqueous prehydrolysate and chlorobenzene or fluorobenzene (1:3 acid/solvent v/v). The organic layer was analyzed at different time intervals, and the results are shown in terms of furfural molar yield.

Chlorobenzene and fluorobenzene showed the highest CMF yields. For this reason, these solvents were also tested for the conversion of DAWN prehydrolysate (xylose oligomers) to furfural. The reactors were loaded with aqueous prehydrolysate, together with chlorobenzene or fluorobenzene in a 1:3 v/v ratio of prehydrolysate/halobenzene. The reaction was screened in batch using a temperature of 80 °C, following previous indications from Mascal and Nikitin.^26^ The reaction proceeded for 4 h, and the organic layer was analyzed at different reaction times. The furfural molar yield obtained in the fluorobenzene or chlorobenzene layer is shown in Figure 12.

The reactors containing fluorobenzene showed consistently higher furfural yield. Also, a maximum yield was observed at different times for each solvent (Figure 12, 1 h for chlorobenzene and 1.5 h for fluorobenzene). After these peak in furfural yields, the concentration of furfural steadily decreased in both solvents due to longer time at 80 °C in the presence of a highly concentrated acidic solution.

### Temperature Screening

A new set of screening experiments were performed to determine the temperature range in which the conversion of glucose DAWN main hydrolysate to CMF is favored. Based on previous screening of solvents (Figure 11), chlorobenzene seemed to be the best candidate for this reaction, and following previous studies from Mascal et al., 60–90 °C was a reasonable range to begin with.^26,28,59^ For this first screening, the reaction time was fixed to 3 h, and reactors at 60 °C showed a relatively slow conversion of glucose to CMF, with an average CMF molar yield of 19.4%. Reactors at temperatures between 70 and 80 °C showed peaks of 87% CMF molar yield, but their reproducibility was lower. These experiments set the basis to later expand it with the inclusion of statistical analysis, which showed that the combined effect of the temperature with other reaction parameters is relevant to draw conclusions (Figure 20, vide infra).

**Figure 13:**
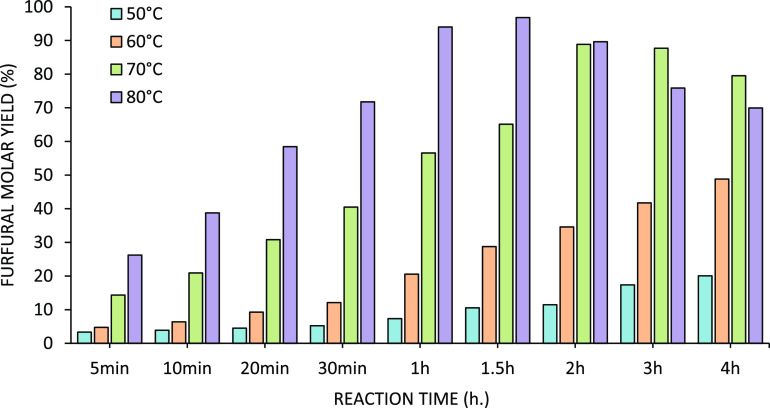
Temperature screening for the conversion of DAWN prehydrolysate, containing xylose, to furfural. The biphasic reactors were loaded with fluorobenzene (organic phase) and DAWN prehydrolysate (xylose oligomers, aqueous phase).

**Figure 14:**
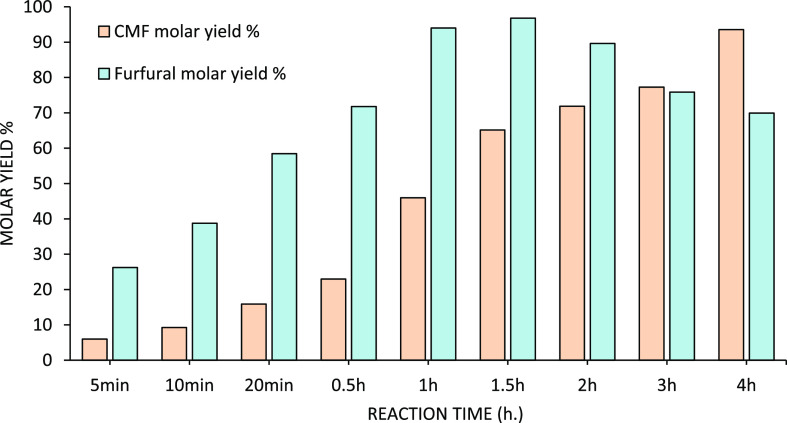
Furfural and CMF molar yields obtained at 80 °C during 4 h. The reaction was carried out in biphasic reactors containing fluorobenzene (organic phase) and DAWN prehydrolysate (aqueous phase). The results are expressed in terms of CMF molar yield (red) and furfural molar yield (blue).

**Figure 15:**
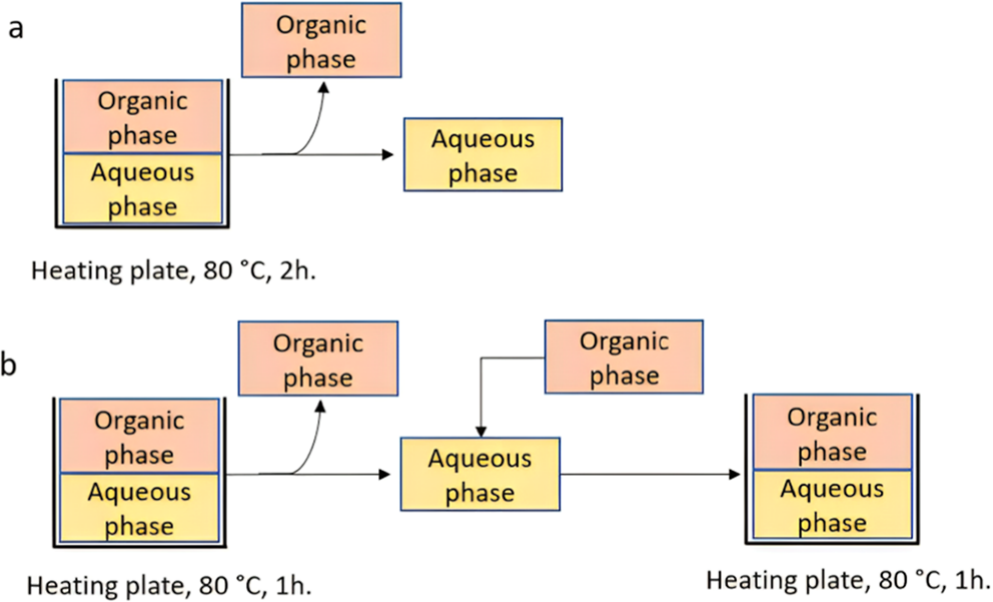
(a) Batch system for the conversion of saccharides to CMF and furfural. In (b), the reaction runs semicontinuous. After every hour, stirring is stopped, the organic layer is separated, and a new organic phase is manually added before resuming the reaction. The total reaction time in batch (a) is divided into blocks of 1 h when running it in the semicontinuous mode (b).

**Figure 16:**
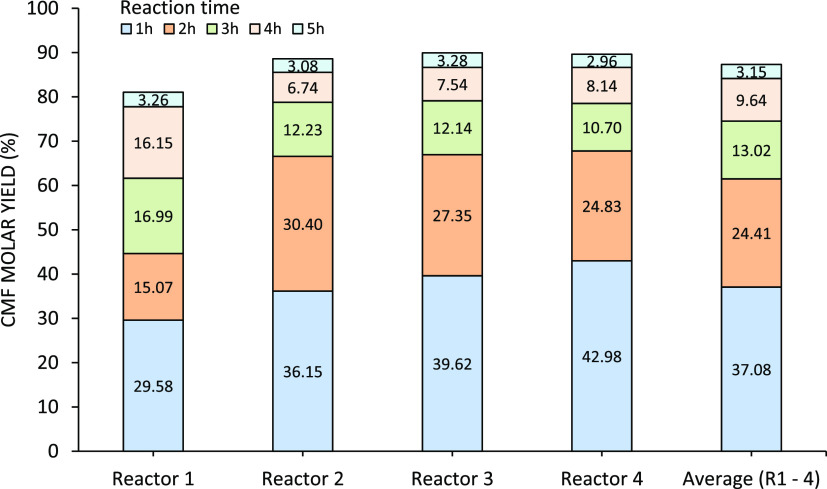
CMF molar yield using the semicontinuous extraction system. Industrial glucose main hydrolysate was fed into the biphasic reactor containing chlorobenzene. The reactor was heated at 80 °C, and the organic phase was refreshed every hour.

**Figure 17:**
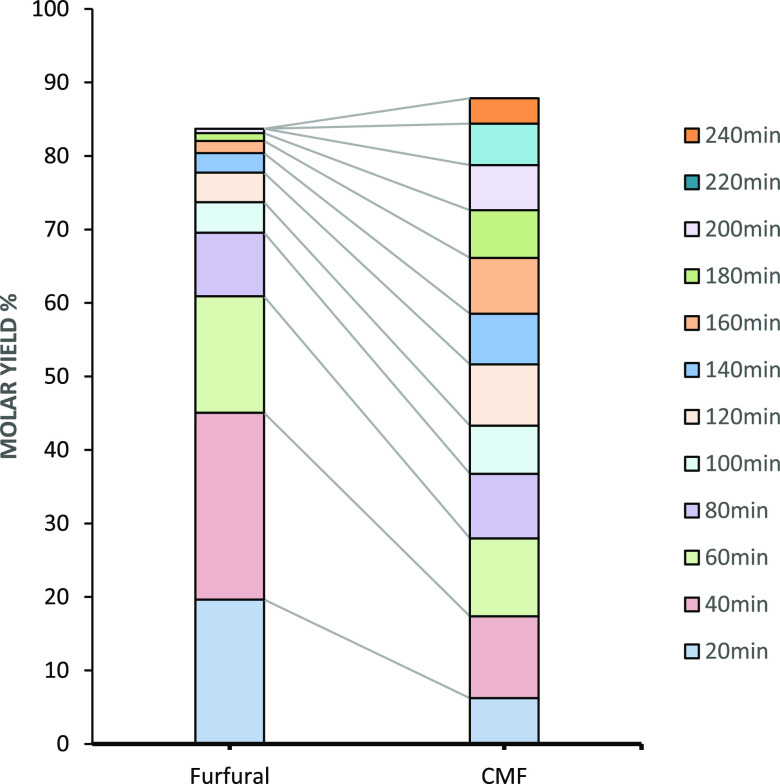
Conversion of prehydrolysate sugars to furfural and CMF. Every 20 min, the organic phase was collected and replaced. The analysis of the organic layer is shown in terms of CMF and furfural molar yield.

**Figure 18:**
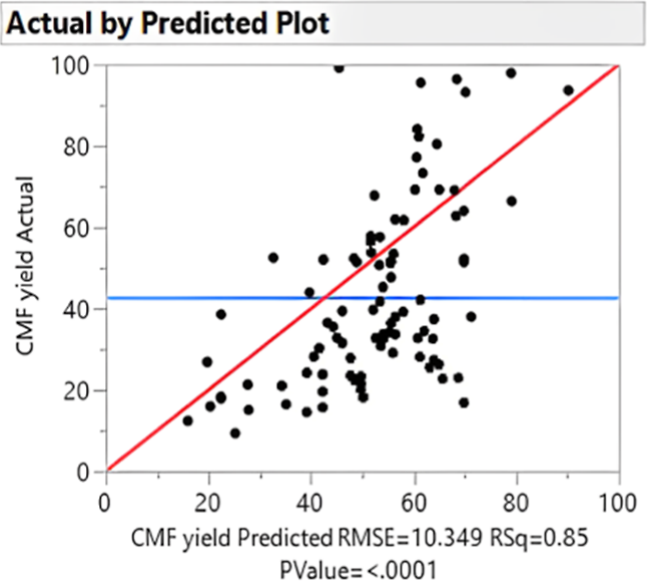
CMF molar yield obtained from the experiments suggested by JMP software versus CMF yield predicted.

**Figure 19:**
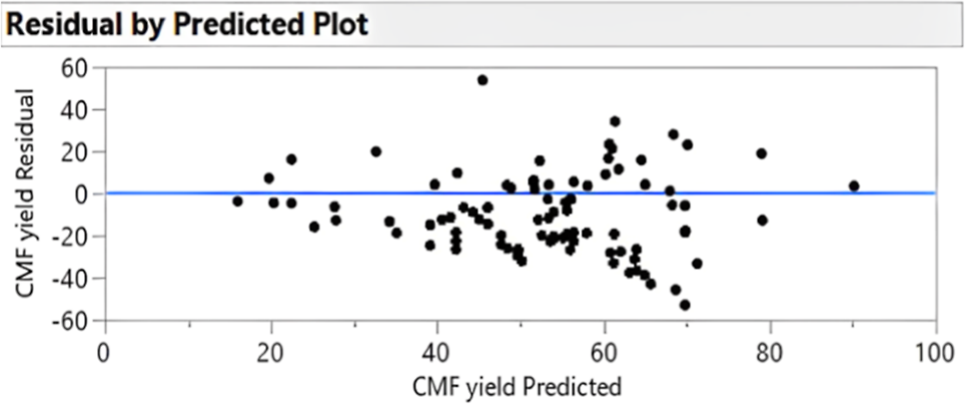
Residuals vs predicted plot. None of the residuals stand out from the basic random pattern of the other residuals.

**Figure 20:**
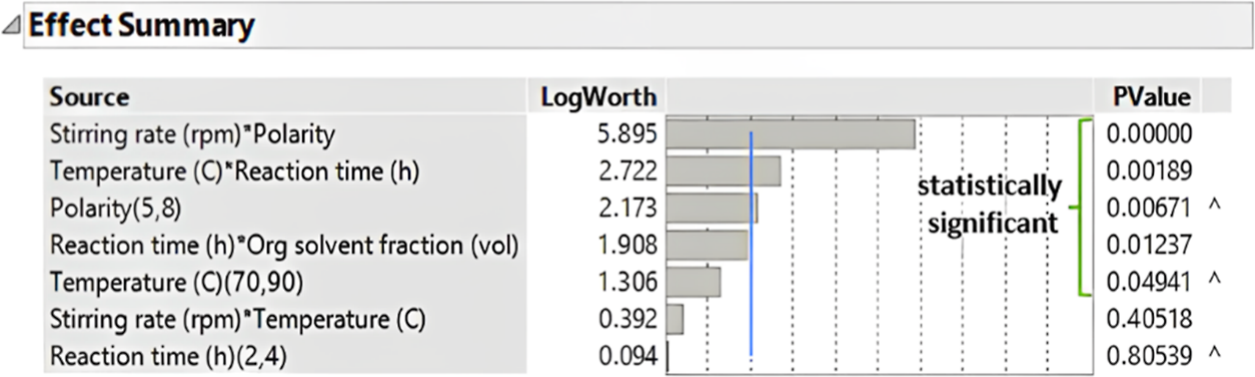
*p*-Values for the individual and combined factors that influence the CMF yield. Terms with *p*-values < 0.05 are shown as statistically significant.

Next, research was focused on finding the temperature to maximize the conversion of DAWN prehydrolysate, containing xylose, to furfural. These experiments were performed using fluorobenzene instead of chlorobenzene as the organic phase. The reason behind lies in the previous solvent screening, in which fluorobenzene showed better prehydrolysate to furfural extraction yields (Figure 12). After black depositions at 90 °C were noted in the previous screening experiments, this temperature was now omitted. For the prehydrolysate screening, temperatures between 50 and 80 °C were investigated, and the results are shown in Figure 13.

Temperatures of 70 and 80 °C showed a higher furfural molar yield, with maxima at 88.8 and 96.8%, respectively. After this, a progressive decline in the furfural concentration in the organic phase was observed. Apparently, at longer contact times, undesired furfural decomposition reactions take place. Temperatures of 50 and 60 °C showed a slow but steady increase in the furfural yield, but the maximum furfural yield is not yet obtained after 4.0 h. In this range of temperature, longer reaction times could increase the final furfural yield, but long contact times are not suitable for scaling up purposes.

A reaction temperature of 80 °C was further investigated, following guidelines of previous observations from Mascal,^26,28,59^ in which 80 °C was consistently recommended for the conversion of glucose (derivatives) to CMF. Moreover, the organic phase was also analyzed for the simultaneous formation of CMF from the glucose present in the prehydrolysate. The hemicellulose can contain up to 10% of the total glucose available in the lignocellulose. This was to see whether the small fractions of glucose, mixed in this prehydrolysate with the xylose, could also be efficiently transformed into CMF at 80 °C. The formation of furfural from xylose was expected to occur at a faster rate compared to the conversion of glucose to CMF. The reactors were loaded with DAWN prehydrolysate and fluorobenzene, and the heating was maintained for 4 h. The CMF and furfural molar yields obtained during this experiment are shown in Figure 14.

Figure 14 shows high furfural yield at shorter reaction times, as compared to the formation of CMF from glucose. 96.8% furfural molar yield was obtained after 1.5 h, while 4.0 h was required to achieve a similar molar yield for CMF (93.6%). After 1.5 h of reaction time, furfural showed decomposition, and the reactor contained black solid depositions. Compared to the conversion of xylose to furfural, in which only one dehydration step is needed, glucose requires longer times. First, glucose must undergo a dehydration step to form HMF, and then, HMF reacts with HCl to go through the nucleophilic substitution required to obtain CMF. From this perspective, the energy required for glucose and xylose to, respectively, convert to CMF or furfural is different, with glucose requiring higher energy. By running this reaction in continuous mode, for example, using a continuous stirred tank reactor (CSTR), the organic phase can be continuously refreshed, so decomposition of furfural at longer reaction times is avoided.

### Reactor Design

The reactor setup was modified to allow for a semicontinuous extraction of the organic solvent (Figure 15). By continuously refreshing the organic phase, the gradient of CMF concentration toward the organic phase increases. Also, removing the CMF from the reactor decreases the contact time with the acidic aqueous phase and with it the amount of undesired reactions with water and HCl.

For the conversion of DAWN main hydrolysate to CMF, after every hour of reaction time at 80 °C, the reactor is cooled to room temperature. Then, the organic layer is separated and analyzed for CMF. New chlorobenzene is added into the reactor to replace the volume taken before, and the heating is resumed at 80 °C for another hour. This semicontinuous extraction is repeated four times to achieve a total reaction time of 5 h. The CMF molar yield obtained at each hour is shown in Figure 16.

The semicontinuous extraction of CMF allowed for a consistent 80–90% total CMF molar yield. From Figure 16, it can be concluded that most of the CMF is formed during the first 2 h, while in the last hour, only small amounts of product were extracted. This suggests that the last hour acts as a washing step of the residual CMF in the aqueous phase, rather than a continuation of the reaction.

By running this reaction in a true continuous manner [e.g., a continuously stirred tank reactor (CSTR)], the reaction time and solvent fraction could potentially be reduced. Brasholz et al. developed the first flow reactor for the continuous production of CMF.^66^ It had two separate nozzles for the aqueous and organic phase. The aqueous phase contained the saccharide dissolved in conc. HCl, and the organic solvent was either dichloromethane (DCM) or dichloroethane. The best results were achieved by running the system with d-fructose in HCl/DCM for a residence time of 1 min at 100 °C.^66^ The challenge in using continuous flow systems for this reaction is the blockages inside the pipelines produced by the formation of humins. Hence, this being a downside, an in-line carbon particle filter and a back-pressure regulator could potentially mitigate the problem.

This semicontinuous reaction system was also applied for the conversion of DAWN prehydrolysate, containing xylose oligomers, to furfural. Similar reaction conditions as for the semicontinuous conversion of glucose to CMF were applied. In this case, the organic solvent was replaced by fluorobenzene, and the reaction was stopped every 20 min to refresh the organic layer. For this study, three reactors containing prehydrolysate and fluorobenzene were heated to 80 °C. The reaction was cooled to room temperature every 20 min to collect the organic phase. New fluorobenzene was added to the reactor, and the reaction was resumed. After 4 h of semicontinuous extraction, the reaction was concluded. The organic phase obtained at every fraction of 20 min was analyzed for CMF and furfural. This reaction was repeated in triplicate, and the average CMF and furfural molar yield obtained at each fraction are shown in Figure 17.

Figure 17 shows a cumulative 61% furfural molar yield after 1 h, while CMF required longer times to achieve similar yields. This quicker furfural formation compared to CMF was already found in previous temperature screening experiments. In this experiment, the total CMF and furfural yields were slightly lower than those seen in previous experiments (Figure 14). However, the semicontinuous removal of the organic phase is still advised. By running the reaction in a continuous reactor, the total volume of solvent is drastically reduced as compared to batch experiments, the residence time of the furfural is minimized, and hence the contact time between furfural and CMF with the acid is also reduced, preventing undesired polycondensation reactions and the formation of insoluble humin material.

### Statistical Analysis

A study of the combined and individual effects of each reaction parameter on the formation of CMF was performed. For this purpose, a statistical analysis software package (JMP, Version 16. SAS Institute Inc., Cary, NC, 1989–2023) was used to create a design of experiments (DoE) and facilitate a consequent statistical analysis. Different ranges of temperature (°C), stirring rate (rpm), reaction time (h), organic solvent fraction (v/v), and solvent permanent dipole force (δ_P_, MPa^1/2^) were combined to generate a design matrix (Table S4) of screening experiments. By selecting interactions between every two of these parameters, the software checked for the possible contribution of individual and combined factors at a time. For instance, temperature and stirring rate impacted the formation of CMF in biphasic systems, so both parameters (combined and individually) were studied. This allowed to isolate the statistically significant parameters and thereby predicting the best combination to ultimately achieve a maximum yield.

The sugar composition of the DAWN main hydrolysate used in this study is shown in Table 3. Based on previous studies from Lane and Mascal,^59^ high starting sugar concentration can detriment the final CMF yields. For this purpose, we chose sugar solutions with glucose concentrations below 10 wt %. Each set of reaction conditions was performed in triplicate, and the average CMF yield obtained was added as input in the design matrix. The range of reaction parameters studied and included in the model is shown in Table 4. The model used is a standard least squares personality of the fit model platform. This model fits a wide spectrum of standard models, and it includes regression, analysis of variance, analysis of covariance, and mixed models. It stands within the models typically used to analyze designed experiments.^67^ The analytic results are supported by compelling dynamic visualization tools, such as profilers, contour plots, and surface plots. These visual displays complement and support the understanding of the model. They enable optimization of several responses simultaneously and exploration of the effect of noise.

**Table 3:** DAWN Main Hydrolysate Sugar Solution

glucose [wt %]	mannose [wt %]	xylose [wt %]	arabinose [wt %]	galactose [wt %]
3.13	0.07	0.90	0.00	0.00

**Table 4:** Range of Reaction Conditions Tested

stirring rate [rpm]	temperature [°C]	reaction time [h]	org. solvent fraction [v/v]	solvent polarity [δ_P_, MPa^1/2^]
600 to 1200	70 to 90	2 to 4	2 to 4	1.4 to 9.0

The coefficient of determination, *R*^2^, showed a correlation between the predicted results and the experimental data. It showed that 85% of the CMF yield variance (Figures 18 and 19) is explained by the variance of the reaction parameters, namely, stirring rate, temperature, reaction time, organic solvent fraction, and solvent polarity. To find the sources of noise, a residual maximum likelihood (REML) analysis was performed. The REML variance component estimates show that 75.5% of the noise is contributed by the whole plot effects. This means that the source of noise mostly comes from small inaccuracies of the equipment, rather than from unknown factors. The noise commonly comes from sources such as the equipment used (e.g., heating distribution across plates).

The effect of the reaction parameters on the CMF yield is shown in Figure 20. For this study, every individual parameter with a *p*-value > 0.05 is not considered statistically significant. This is because the contribution of these parameters to the CMF yield was by more than 5% of probability due to random effects. An exception of this rule occurs when an individual parameter with a *p*-value > 0.05 is contained in a combined factor with a *p*-value < 0.05. For instance, this was found in the reaction, which has a *p*-value of 0.8054 (Figure 20), but in combination with the temperature, it has a *p*-value < 0.05.

In the effect summary in Figure 20, the combined effect of the stirring rate with the solvent polarity showed the biggest impact on the CMF yield. This indicates that combined changes in these two parameters are significant for the outcome of the reaction. It also suggests that the ability of the organic layer, determined by its polarity, to extract and shelter the CMF from the aqueous phase should be taken into consideration. The stirring rate also impacts the CMF extraction by creating efficient mixing and contact between the two immiscible phases. The impact of this combined effect in the CMF yield is aligned with previous studies,^59^ which remarks the requirement of an efficient CMF and HMF extraction from the aqueous phase to achieve high CMF yields. The combined effect of temperature and reaction time shown in Figure 20 was also remarkable and statistically significant for the course of reaction, which also aligned with previous research from Lane et al.^59^ While an optimal temperature range allowed for high CMF yields, this in combination with the reaction time became statistically more relevant. With experiments at the extremes of these parameters, e.g., entries 24 and 26 of Table S4 in the Supporting Information, it was shown that heating at 70 °C for 4 h or 90 °C for 2 h provided similar CMF yields (52.5 and 53.7%, respectively). The organic solvent fraction is relevant only in combination with the reaction time. This could suggest that for short reaction times and low saccharide conversion, low amounts of organic solvent could suffice to shelter all the CMF formed. While for longer reaction times and higher final CMF amounts, higher volumes of organic solvents might be required.

**Figure 21:**
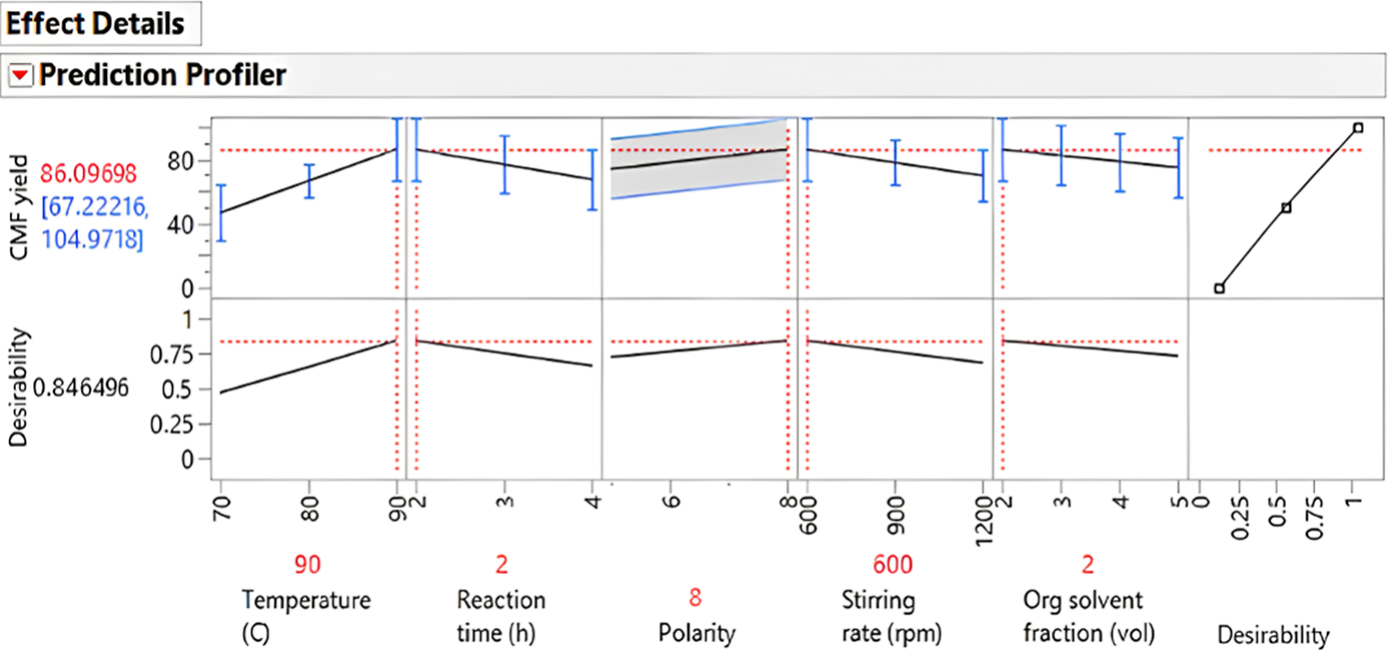
Prediction profiler to maximize the CMF yields. The highest yield predicted from the input data of the experiments is 86.1%. To achieve this yield, the reaction conditions recommended are a temperature of 90 °C, 2 h reaction time, an organic solvent polarity of 8.0 δ_P_, MPa^1/2^, a stirring rate of 600 rpm, and an organic solvent fraction of 1:2 (aqueous phase/organic layer, v/v).

The prediction profiler in Figure 21 shows the effect of each individual parameter on the final CMF yield. It also suggests the reaction conditions to achieve high CMF yields (Figure 21 in red). It includes a desirability profiling, in which we specified the desirability to maximize CMF yield based on each response or reaction parameter, namely, temperature, reaction time, stirring rate, and organic solvent fraction and polarity. The overall desirability for all responses is defined as the geometric mean of the desirability functions for the individual responses.

The prediction profiler showed lower CMF yields at very high stirring rates. Although CMF is less polar than its hydroxy analogue, HMF, it is still a relatively polar compound. For this reason, at very high stirring rates, CMF in the organic phase could pass to the aqueous phase and react with the acid and water present in it to form HMF, levulinic acid, and polycondensation products (humins), lowering the final CMF yield. It also suggests that higher temperatures (90 °C) and short reaction times (2 h) are best to achieve high CMF yields. From this profiler, temperature had the biggest impact on the CMF yield, but overall, either in combination with other factors or individually, the organic solvent polarity had the biggest statistical significance during the course of the reaction.

The reaction conditions recommended by the prediction profiler were replicated in the laboratory. The conversion of DAWN main hydrolysate (sugar composition in Table 3) was tested following the recommended reaction parameters; a temperature of 90 °C, 2 h reaction time, a stirring rate of 600 rpm, and a ratio of 1:2 (aqueous phase/organic layer, v/v) were chosen. The polarity of the organic solvent was slightly higher, corresponding to that of *o*-difluorobenzene (MPa^1/2^ = 9.0). After 2 h, the reaction was concluded, the organic phase was collected, and the aqueous phase was washed at room temperature using the same starting ratio of difluorobenzene (2 mL of organic phase). This washing step was repeated twice, and all the organic layers were combined and analyzed for CMF. The analysis of these combined organic layers showed a total CMF yield of 96.7%.

### Environmental Impact and Evaluation

The environmental impact of this integrated process, including the biomass saccharification and biobased furanic production, is widely determined by the nature of the feedstock, the capacity to recycle the acid and the halogenated solvents, and the outlet for the lignin and humin coproducts. A conceptual process design and techno-economic evaluation study was performed by Process Design Centre, in Breda, The Netherlands. This assessment took into account the different streams (inlets and outlets) involved in the production of CMF and furfural from wood biomass.

The biomass was provided by the Dutch forestry commission, Staatsbosbeheer, which controls and provides sustainable feedstock. They divide limited sections of the forestry areas within interested parties and supply them with wood biomass at different time lapses to continuously reforest and make a net zero deforestation of the land.

The aqueous HCl/H_2_O streams are directed to a common HCl reconcentration and absorption section. This section was developed for the DAWN saccharification process at the pilot scale and effectively tested in pilot trials. This absorption uses two HCl distillation columns operated at different pressures (dual pressure distillation) to circumvent the HCl/water azeotrope and two concentrator absorbers. HCl/H_2_O pressure swing distillation is performed at 6 bar for the HP (high-pressure) column and 0.2 bar for the LP (low-pressure) column. In the HP column, a high concentration of HCl at the top and HCl/H_2_O with a composition close to but above (in terms of HCl concentration) the azeotrope at the bottom are obtained. Due to the pressure effect, the HCl/H_2_O composition from the bottom of the HP column is under the azeotrope composition at low pressure. This stream is treated in the vacuum column, where water effluent is from the top. The concentrated HCl/H_2_O from the bottom is sent back to the HP column. Absorption of HCl is performed in two falling-film absorbers. In one, 42 wt % HCl is prepared from part of the HCl–water stream coming from the final decanter of the CMF reactor section and HCl rich gas from the top of the HP column. In the other falling-fil absorber, 37 wt % HCl is prepared from the HCl-rich gas from the top of the HP column and a mixture of the part of the HCl–water stream coming from the last decanter of the furfural and CMF reaction section.

Humins are formed inside the CMF and furfural reactors as a consequence of polycondensation reactions between the saccharides and dehydration intermediates under acidic conditions. They frequently stay insoluble in the aqueous phase (bottom layer of the biphasic system) and thus can be re filtered out from the HCl/H_2_O stream before this stream is circulated to the dual distillation to recycle it.

The major environmental impact of the process is related to the use of halogenated organic solvents, such as chlorobenzene and fluorobenzene. It is widely known that their use must be limited, and when alternatives are found, it is strongly recommended to pursue the scale-up of future technologies with other similar compounds.^68,69^ During this study, few alternatives to halogenated solvents were also tested (Figure 11). However, the presence of a halogen in the organic solvent greatly improves the selectivity of extraction toward CMF and furfural. Due to the high selectivity of these solvents to solely extract CMF and furfural from the aqueous acidic solution, this also allows for a simple distillation to separate the products from the solvent and fully recycle the solvent back to the CMF and furfural reactor. The small amounts of these solvents still present in the aqueous layer after phase separation are recovered in the dual distillation, acting as a doubled-layered protection to avoid their emission to the atmosphere.

The lignin required washing with water to recover the acid present in it. The water washings of the lignin provide a diluted acidic solution that is used to wash new batches of lignin and increase the HCl concentration in it. When the acid concentration reaches 30 wt %, it is recirculated to the HCl absorber for the production of 42 wt % HCl, as described in the Materials and Method section. Lignin produced in this pilot tests was tested to replace bitumen in asphalt, with the world’s first test road made with lignin produced by DAWN technology in The Netherlands in 2021.^70,71^

## Conclusions

Biomass valorization and low-energy saccharification technologies need to be cost-effective and commercially attractive to reduce the strong dependence of fossil feedstock based technologies. This study presents a solution for low-temperature and ambient pressure HCl-based biorefineries, whose main challenges are found in the downstream acid–sugar separation. This was achieved by integrating the direct conversion of the hydrolyzed sugars in HCl into furanic platform molecules, like CMF and furfural. The in situ extraction of these furanics into an immiscible organic solvent allowed us to obtain CMF and furfural, from C_6_ and C_5_ sugars, respectively, in 80–90% molar yield. The downstream separation of these furanics from the organic solvent is done by distillation, in which the acid and aqueous phases are not present. The most influential reaction parameters in the conversion of C_6_ saccharides to CMF were studied separately and later combined. It was concluded that the combined factors of stirring rate with the polarity of the organic solvent, followed by the temperature with the reaction time, had the biggest impact on the furanics-isolated yields. The best reaction conditions were found to be 90 °C, 2 h reaction time, an organic solvent polarity of 8.0 (δ_P_, MPa^1/2^), a stirring rate of 600 rpm, and an organic solvent fraction of 1:2 (aqueous phase/organic layer, v/v). This altogether allowed us to achieve 96.70% CMF molar yield from the C_6_ hydrolyzed sugars in HCl solutions from the saccharification of lignocellulosic biomass.

## Supporting Information Available

Characterization methods; complete description corresponding to the DAWN saccharification technology; and design of experiments corresponding to the statistical analysis section (PDF)
