# Supplementary material for: Production and Downstream Integration of 5-(Chloromethyl)furfural from Lignocellulose
Source: ACS Sustain Chem Eng. 2023 Dec 1;11(49):17492–509. doi: 10.1021/acssuschemeng.3c05525 (PMC10716901; doi:10.1021/acssuschemeng.3c05525)
Supplement: Supplementary file 1 — sc3c05525_si_001.pdf [file sc3c05525_si_001.pdf]

Supporting information

Solving the Biorefinery Puzzle: Downstream Integration for Biomass Valorization

Jorge Bueno Moron[a,],[b], Gerard van Klink[b], and Gert-Jan M. Gruter[a], [b]*

[a] Van‘t Hoff Institute for Molecular Sciences, University of Amsterdam, Science Park 904, 1090 GD Amsterdam, The Netherlands

E-mail: g.j.m.gruter@uva.nl, j.buenomoron@uva.nl

[b] Avantium Chemicals BV, Zekeringstraat 29, 1014 BV Amsterdam, The Netherlands

Number of pages: 11

Number of figures: 1

Number of tables: 4

Characterization Methods

Ion Exchange Chromatography. Ion exchange chromatography (IC) was used for the quantification of soluble monosaccharides, namely: arabinose, rhamnose, galactose, glucose, xylose, mannose and fructose in HCl using anion exchange chromatography coupled with a pulsed amperometric detector (PAD). The analysis was performed on a Dionex ICS-5000 system, equipped with and auto sampler, dual pump, a detection compartment with a heated column compartment and an electrochemical detector with disposable gold electrode. The separation was achieved with an CarboPac PA1 Analytical Anion Exchange Column (2 x 250 mm). 10 μL sample, containing 1 mg · mL $^{-1}$ mg/mL melibiose as internal standard, was injected and separated using a gradient. The mobile phase (flow rate: 0.4 mL · min $^{-1}$, 30 ˚C) consisted of a mixture of MilliQ water (solvent A) and 0.1 M sodium hydroxide in MilliQ water (solvent B). The initial percentage of solvent B was 0% held for 15 minutes and then increased linearly to 5% at 30 minutes after

which it was increased to 100% in 0.1 minute and held for 4.9 minutes. The initial solvent B concentration was restored to the initial conditions in 0.1 minutes and maintained for 5.9. Between runs, the system was flushed with MilliQ for 8 minutes given a total run time of 50 minutes.

High Performance Liquid Chromatography. High Performance Liquid Chromatography (HPLC) was used to determine the concentration of sugar degradation products. HPLC was performed on an Agilent 1260 Infinity II system with 1260 series refractive index (RID) and diode-array (DAD WR) detectors. The column was an Aminex HPX-87H (300 × 7.8 mm; dp 9 μm). 7 μL sample was injected and separated using 5 mM H_2SO_4 in MilliQ water (flow rate: 0.6 mL ·/ min $^{-1}$, 60 ˚C) as the mobile phase. CMF, HMF and furfural were detected at 230 nm whereas RID was used to analyse acetic acid, formic acid, and levulinic acid.

Gas Chromatography. The final product yield of CMF was identified using gas an Agilent 5975C gas chromatograph (GC) with triple axis mass selective detector (MSD). The system was equipped with two spitless injectors, an autosampler, and a headspace-autosampler. 0.5 μL of a 1 mg ·mL $^{-1}$ sample solution in methanol was injected on a Agilent J&W DB624 column (20 m x 0.18 mm x 1 μm) using Helium as the carrier gas (1.2 1 mg · mL $^{-1}$, 1:25 split ratio, 250 ˚C inlet temperature). Separation was achieved using a temperature gradient starting at 50 ˚C for 2 minutes after which

the temperature increased to 170 ˚C with a 15 ˚C ·min $^{-1}$ ramp immediately followed by a second ramp of 5 ˚C · min $^{-1}$to 250 ˚C after which the temperature was held constant for 10 minutes having a total runtime of 36 minutes. Mass spectra were obtained at full scan mode. Compounds were ionized using electron ionization at an electron energy of 70 eV, source temperature of 230 ˚C and quadrupole temperature of 150 ˚C. Agilent Mass Hunter program with NIST library 11 and a reference standard were used for identification.

CMF and degradation products were quantified with a Thermo Scientific Trace 1310 GC equipped with a TriPlus RSH autosampler for liquid injection, two SSL injectors and two FID detectors. In this project the front SSL injector and the front FID channel are used. 0.5 μL of a 1 mg · mL $^{-1}$sample solution in chlorobenzene were injected on a Agilent J&W DB-624 UI (30 m × 0.25 mm × 1.4 μm) using Helium as the carrier gas (2.5 mL · min $^{-1}$, 1:100 split ratio, 250 ˚C inlet temperature). 1 mg · mL $^{-1}$1,4-dioxane was used as an internal standard. The temperature gradient started with an initial temperature of 70 ˚C that was held constant for 8 minutes after which the temperature increased to 130 ˚C with a 15 ˚C · min $^{-1}$ ramp and a second ramp of 40 ˚C · min $^{-1}$ to 250 ˚C that was held constant for 5 minutes resulting in a total run time of 20 minutes.

^{1}H-NMR Spectroscopy. ^{1}H-NMR analysis was done on a Bruker Avance III HD 600 including a Cryo Probe Inverse Triple Resonance 5 mm with Z-gradient; 3 independent channels and lock channel; 1H, 2H, 13C and 15N; Cryo Cooling Unit 3m. The system was operated using Topspin 3.6 and data was processed with MestReNova. 15 mg sample was dissolved in 0.7 mL deuterated chloroform ($CDCl_3$).

Results and discussion

Dawn saccharification technology®

The DAWN-modified Bergius process is summarized in nine steps:

1. Wood de-barking, chipping and drying
2. Hydrolysis
3. HCl/H_2O evaporation
4. Oil-sugar separation
5. Post-hydrolysis
6. HCl distillation and absorption
7. Sugar purification

8. Lignin drying
9. Lignin pelletization

The process starts unloading bundles of wood logs to a log receiving deck with a stone separator. A conveyor feeds the logs into two debarking units. 1% wood losses are assumed in the screening section, besides 12% of bark.

The wood chips are then dried in a belt dryer to reduce the amount of water and ensure proper operation of the pre-hydrolysis. In the product feed module, the material is fed continuously by a distribution screw across the entire width of the dryer belt. The distribution screw ensures uniform layer thickness across the entire width. The product on the belt is warmed-up and dried by hot air passing through it. The drying air is heated in air heat exchangers. Drying air with a maximum temperature of 110 ˚C is directed from top to bottom through the belt dryer. The dried product is discharged by a screw to a belt conveyor and sent to the dried wood storage vessel.

The dried wood chips are loaded into reactors lined with PVC. Each reactor has a bottom outlet with annular sieves below for the product off-take, and a Hastelloy valve which is HCl resistance.

The reactor system is designed as a simulated moving bed of seven identical reactors in two parallel trains. Each reactor is operated in switch mode, in which 7 phases of 8 hours can be distinguished:

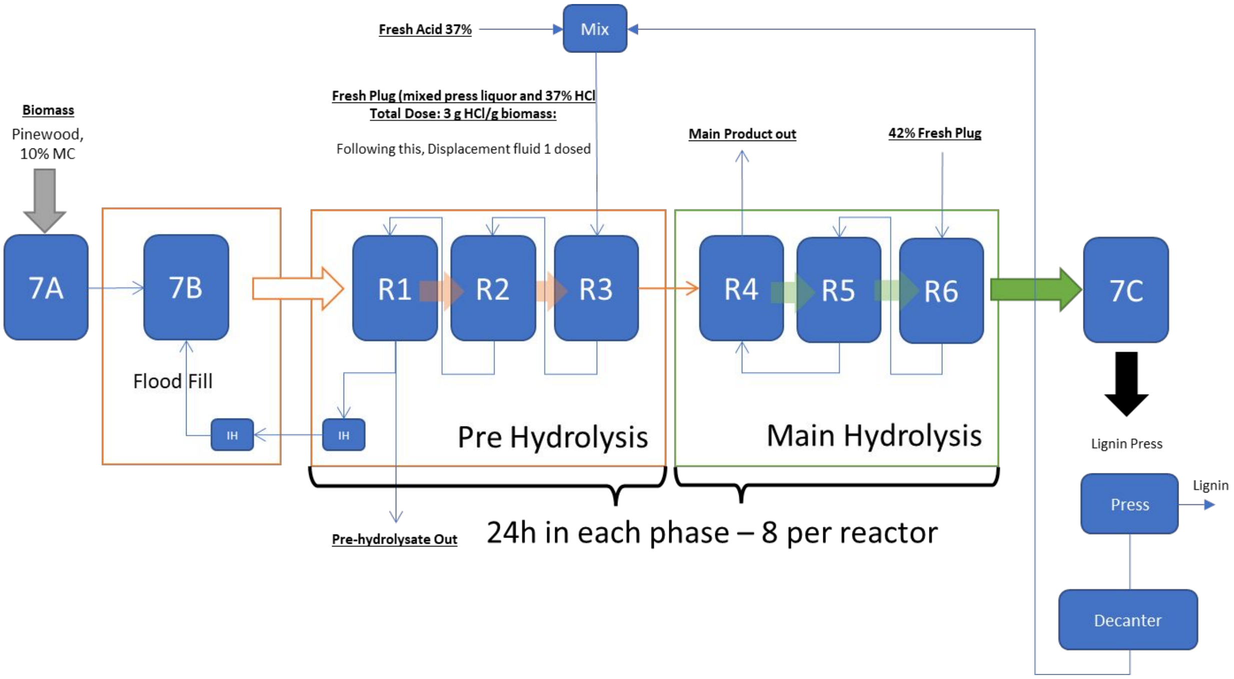

Figure S1. Block flow diagram of the simulated moving bed hydrolysis. The biomass is loaded into the first reactor (7A), the reactor is quickly filled with intermediate hydrolysate (HCl 37% with some hemicellulosic sugars in it, 7B). Then, this reactor becomes R1 and the first block of 8 hours starts. After each block of 8 hours, every R(X) reactor becomes R(X+1).

Table S1. Description of the hydrolysis stages.

Reactor Phase	Phase time (h)	Description
7A	3.5	Reactor is filled with wood chips of 10% moisture content.
7B	0.5	Reactor is flood filled (i.e. quickly) from the bottom with intermediate hydrolysate

1A	8	Reactor top is connected to bottom of R+1. Pre hydrolysate is collected from the bottom of R1
1B		Reactor top is connected to bottom of R+1. Intermediate hydrolysate is collected from the bottom of R1. Volume collected to ensure Intermediate hydrolysate tank level stays constant.
2	8	Reactor top is connected to bottom of R+1, reactor bottom is connected to top of R-1.
3A	8	Lignin press liquor is pumped to the reactor top, reactor bottom is connected to top of R-1.
3B		37% HCl is pumped to reactor top
3C		Displacement fluid is pumped to reactor top, reactor bottom is connected to top of R-1. Displacement fluid pushes acid out of the reactor.
4A	8	Displacement fluid is collected from reactor top, reactor bottom is connected to bottom of R+1. Displacement fluid is pushed out of the reactor.
4B		Main hydrolysate is collected from reactor top, reactor bottom is connected to bottom of R+1.
5	8	Reactor top is connected to bottom of R+1, reactor bottom is connected to bottom of R-1.
6A	8	42% HCl is pumped to reactor top, reactor bottom is connected to bottom of R-1.
6B		Displacement fluid is pumped to reactor top, reactor bottom is connected to top of R-1. Displacement fluid pushes acid out of the reactor.
7C	4	Reactor is emptied (residual lignin + retained acid + displacement fluid). Resets to 7A.

After completion of the hydrolysis, the reactor is emptied by fluidization of the suspended lignin particles with displacement fluid, and this suspension is transported to a Hastelloy screw press, where the solids are mechanically pressed to reduce the liquid content. The recovered hydrolysate and the displacement fluid from inside the lignin are separated in a decanter, after which the hydrolysate is sent to a storage tank. From this tank, the press liquor is recycled to the hydrolysis reactors (Phase 3A). The displacement fluid is recycled, and a purge is applied to avoid build-up of impurities in the displacement fluid.

Table S2. Pre-hydrolysis performance for Aspen woodchips feedstock.

	Saccharification yield %	C_6 sugar to HMF molar yield %	C_5 sugar to furfural molar yield %
Glucose	6	0.12	-
Xylose	100	-	0.79
Arabinose	100	-	0.79
Galactose	100	0.12	-
Mannose	100	0.12	-
Rhamnose	100	0.12	-
Methanol	100	-	-
Acetic acid	100	-	-

Table S3. Main-hydrolysis performance for Aspen woodchips feedstock.

	Saccharification yield %	C_6 sugar to HMF molar yield %	C_5 sugar to furfural molar yield %
Glucose	100	0.22	-
Xylose	-	-	9.5
Arabinose	-	-	9.5
Galactose	-	0.22	-
Mannose	-	0.22	-
Rhamnose	-	0.22	-

Statistical analysis

Table S4. Experimental design matrix and run results (DoE).

Entry	Stirring rate (rpm)	Temperature (˚C)	Reaction time (h.)	Org. solvent fraction (v/v)	Solvent polarity (δ_P, $MPa^{1/2}$)	RUN RESULTS: CMF yield (%)
1	600	70	2	2	1.4 (Toluene)	26.88
2	600	70	4	4	1.4 (Toluene)	52.49
3	600	70	3	3	9.0 (*o*-Difluorobenzene)	41.72
4	1200	70	4	3	1.4 (Toluene)	69.23
5	1200	70	2	4	1.4 (Toluene)	52.31
6	1200	70	2	2	9.0 (*o*-Difluorobenzene)	32.79
7	600	70	2	4	9.0 (*o*-Difluorobenzene)	36.49
8	600	70	4	2	9.0 (*o*-Difluorobenzene)	53.43
9	600	70	3	2	1.4 (Toluene)	38.56
10	600	90	4	2	1.4 (Toluene)	28.17
11	600	90	2	3	1.4 (Toluene)	51.21
12	600	90	4	4	9.0 (*o*-Difluorobenzene)	97.88
13	1200	70	4	2	1.4 (Toluene)	42.10
14	1200	70	2	3	1.4 (Toluene)	39.68
15	1200	70	4	4	9.0 (*o*-Difluorobenzene)	39.17
16	1200	90	3	2	1.4 (Toluene)	51.43
17	1200	90	4	4	1.4 (Toluene)	69.08
18	1200	90	2	4	9.0 (*o*-Difluorobenzene)	32.73
19	1200	90	2	2	1.4 (Toluene)	66.41
20	1200	90	4	2	9.0 (*o*-Difluorobenzene)	20.12
21	1200	90	3	4	1.4 (Toluene)	64.01
22	600	90	2	2	9.0 (*o*-Difluorobenzene)	93.60
23	600	90	4	3	1.4 (Toluene)	35.52
24	600	90	2	4	1.4 (Toluene)	53.74
25	600	70	2	2	1.40 (Toluene)	26.88
26	600	70	4	4	1.40 (Toluene)	52.49
27	600	70	3	3	9.00 (*o*-Difluorobenzene)	41.72

28	1200	70	4	3	1.40 (Toluene)	69.23
29	1200	70	2	4	1.40 (Toluene)	52.31
30	1200	70	2	2	9.00 (*o*-Difluorobenzene)	32.79
31	600	70	2	4	9.00 (*o*-Difluorobenzene)	36.49
32	600	70	4	2	9.00 (*o*-Difluorobenzene)	53.43
33	600	70	3	2	1.40 (Toluene)	38.56
34	600	90	4	2	1.40 (Toluene)	28.17
35	600	90	2	3	1.40 (Toluene)	51.21
36	600	90	4	4	9.00 (*o*-Difluorobenzene)	97.88
37	1200	70	4	2	1.40 (Toluene)	42.10
38	1200	70	2	3	1.40 (Toluene)	39.68
39	1200	70	4	4	9.00 (*o*-Difluorobenzene)	39.17
40	1200	90	3	2	1.40 (Toluene)	51.43
41	1200	90	4	4	1.40 (Toluene)	69.08
42	1200	90	2	4	9.00 (*o*-Difluorobenzene)	32.73
43	1200	90	2	2	1.40 (Toluene)	66.41
44	1200	90	4	2	9.00 (*o*-Difluorobenzene)	20.12
45	1200	90	3	4	1.40 (Toluene)	64.01
46	600	90	2	2	9.00 (*o*-Difluorobenzene)	93.60
47	600	90	4	3	1.40 (Toluene)	35.52
48	600	90	2	4	1.40 (Toluene)	53.74
49	1200	90	4	4	6.30 (*o*-Dichlorobenzene)	82.23
50	1200	90	3	3	5.10 (*m*-Dichlorobenzene)	80.41
51	1200	90	2	3	5.10 (*m*-Dichlorobenzene)	93.15
52	1200	70	4	3	6.30 (*o*-Dichlorobenzene)	61.72
53	1200	70	3	4	5.10 (*m*-Dichlorobenzene)	50.67
54	1200	70	3	2	6.30 (*o*-Dichlorobenzene)	57.75
55	1200	70	3	4	6.30 (*o*-Dichlorobenzene)	56.50

56	1200	70	3	2	8.20 (1,3-Dichloropropane)	51.47
57	1200	70	4	4	6.30 (*o*-Dichlorobenzene)	73.27
58	600	90	4	2	8.20 (1,3-Dichloropropane)	62.75
59	600	90	3	2	6.30 (*o*-Dichlorobenzene)	52.21
60	600	90	3	4	5.10 (*m*-Dichlorobenzene)	26.23
61	600	70	3	3	6.30 (*o*-Dichlorobenzene)	52.03
62	600	70	2	2	6.30 (*o*-Dichlorobenzene)	43.93
63	600	70	4	4	6.30 (*o*-Dichlorobenzene)	32.74
64	1200	90	2	3	6.30 (*o*-Dichlorobenzene)	96.36
65	900	80	2	5	9.00 (*o*-Difluorobenzene)	67.78
66	900	80	3	3	9.00 (*o*-Difluorobenzene)	69.22
67	1200	80	2	5	9.00 (*o*-Difluorobenzene)	99.13
68	1200	80	4	3	4.25 (1,2,4-Trichlorobenzene)	77.16
69	1200	90	4	3	9.00 (*o*-Difluorobenzene)	57.57
70	900	80	4	3	9.00 (*o*-Difluorobenzene)	84.06
71	900	80	3	5	1.40 (Toluene)	18.16
72	900	80	2	3	1.40 (Toluene)	21.55
73	900	80	2	5	4.30 (Chlorobenzene)	39.40
74	900	80	2	5	5.50 (Bromobenzene)	27.82
75	900	80	3	5	4.30 (Chlorobenzene)	45.25
76	900	80	3	3	6.10 (Fluorobenzene)	37.90
77	900	80	3	3	6.10 (Fluorobenzene)	61.90
78	900	80	3	3	5.50 (Bromobenzene)	51.67
79	900	80	3	3	5.50 (Bromobenzene)	47.67
80	900	80	2	5	6.10 (Fluorobenzene)	22.38
81	900	80	2	5	1.40 (Toluene)	19.58
82	900	80	2	5	1.40 (Toluene)	23.79

83	600	70	2	3	4.30 (Chlorobenzene)	15.07
84	600	70	2	3	1.40 (Toluene)	12.32
85	600	70	3	3	5.50 (Bromobenzene)	24.19
86	600	70	2	5	4.30 (Chlorobenzene)	15.92
87	600	70	3	5	5.50 (Bromobenzene)	14.51
88	600	70	2	5	6.10 (Fluorobenzene)	21.29
89	600	70	2	5	5.50 (Bromobenzene)	9.34
90	600	70	3	3	4.30 (Chlorobenzene)	21.01
91	600	70	3	5	1.40 (Toluene)	17.91
92	600	70	3	5	6.10 (Fluorobenzene)	30.22
93	600	70	2	3	6.10 (Fluorobenzene)	16.43
94	600	70	3	3	1.40 (Toluene)	18.25
95	900	80	2	3	5.50 (Bromobenzene)	34.14
96	900	80	2	3	6.10 (Fluorobenzene)	29.12
97	900	80	3	5	6.10 (Fluorobenzene)	33.65
98	900	80	2	3	1.40 (Toluene)	23.28
99	900	80	2	3	4.30 (Chlorobenzene)	30.82
100	900	80	2	5	4.30 (Chlorobenzene)	31.53
101	900	80	3	3	4.30 (Chlorobenzene)	32.81
102	900	80	3	3	5.50 (Bromobenzene)	36.39
103	900	80	3	5	4.30 (Chlorobenzene)	33.60
104	900	80	2	5	1.40 (Toluene)	15.70
105	900	80	2	5	5.50 (Bromobenzene)	23.52
106	900	80	3	5	4.30 (Chlorobenzene)	32.47
107	1200	90	2	3	4.30 (Chlorobenzene)	37.97
108	1200	90	3	5	6.10 (Fluorobenzene)	25.47
109	1200	90	2	5	6.10 (Fluorobenzene)	28.11
110	1200	90	3	3	1.40 (Toluene)	16.85
111	1200	90	3	3	4.30 (Chlorobenzene)	22.72
112	1200	90	2	5	5.50 (Bromobenzene)	34.47
113	1200	90	3	3	5.50 (Bromobenzene)	27.32

114	1200	90	2	5	4.30 (Chlorobenzene)	32.57
115	1200	90	2	3	6.10 (Fluorobenzene)	22.95
116	1200	90	3	5	5.50 (Bromobenzene)	37.40
